# Extracellular vesicles from human cardiac stromal cells up-regulate cardiomyocyte protective responses to hypoxia

**DOI:** 10.1186/s13287-024-03983-y

**Published:** 2024-10-12

**Authors:** Andreas Czosseck, Max M. Chen, Chuan-Chih Hsu, Gleb Shamrin, Annette Meeson, Rachel Oldershaw, Helen Nguyen, Dora Livkisa, David J. Lundy

**Affiliations:** 1 Graduate Institute of Biomedical Materials & Tissue Engineering, College of Biomedical Engineering, Taipei Medical University, 301 Yuantong Road, Taipei, 235603 Taiwan; 2 Department of Surgery, School of Medicine, College of Medicine, Taipei Medical University, 250 Wuxing Street, Taipei, 110 Taiwan; 3 Division of Cardiovascular Surgery, Department of Surgery, Taipei Medical University Hospital, 250 Wuxing Street, Taipei, 110 Taiwan; 4 Cancer Molecular Biology and Drug Discovery, College of Medical Science and Technology, Taipei Medical University, 250 Wuxing Street, Taipei, 110 Taiwan; 5 Biosciences Institute, Newcastle University, Newcastle upon Tyne, NE1 3BZ UK; 6 Department of Musculoskeletal and Ageing Science, Institute of Life Course and Medical Sciences, Faculty of Health and Life Sciences, University of Liverpool, William Henry Duncan Building, 6 West Derby Street, Liverpool, L7 8TX UK; 7 International PhD Program in Biomedical Engineering, College of Biomedical Engineering, Taipei Medical University, 301 Yuantong Road, Taipei, 235603 Taiwan; 8 Center for Cell Therapy, Taipei Medical University Hospital, 250 Wuxing Street, Taipei, 110 Taiwan; 9 College of Biomedical Engineering, 301 Yuantong Road, Taipei, 235605 Taiwan

**Keywords:** Apoptosis, Mesenchymal stromal cell, Exosome, RNA-sequencing, miRNA, Multi-omics

## Abstract

**Background:**

Cell therapy can protect cardiomyocytes from hypoxia, primarily via paracrine secretions, including extracellular vesicles (EVs). Since EVs fulfil specific biological functions based on their cellular origin, we hypothesised that EVs from human cardiac stromal cells (CMSCLCs) obtained from coronary artery bypass surgery may have cardioprotective properties.

**Objectives:**

This study characterises CMSCLC EVs (C_EVs), miRNA cargo, cardioprotective efficacy and transcriptomic modulation of hypoxic human induced pluripotent stem cell-derived cardiomyocytes (iPSC-CMs). C_EVs are compared to bone marrow mesenchymal stromal cell EVs (B_EVs) which are a known therapeutic EV type.

**Methods:**

Cells were characterised for surface markers, gene expression and differentiation potential. EVs were compared for yield, phenotype, and ability to protect hiPSC-CMs from hypoxia/reoxygenation injury. EV dose was normalised by both protein concentration and particle count, allowing direct comparison. C_EV and B_EV miRNA cargo was profiled and RNA-seq was performed on EV-treated hypoxic hiPSC-CMs, then data were integrated by multi-omics. Confirmatory experiments were carried out using miRNA mimics.

**Results:**

At the same dose, C_EVs were more effective than B_EVs at protecting CM integrity, reducing apoptotic markers, and cell death during hypoxia. While C_EVs and B_EVs shared 70–77% similarity in miRNA content, C_EVs contained unique miRNAs, including miR-202-5p, miR-451a and miR-142-3p. Delivering miRNA mimics confirmed that miR-1260a and miR-202/451a/142 were cardioprotective, and the latter upregulated protective pathways similar to whole C_EVs.

**Conclusions:**

This study demonstrates the potential of cardiac tissues, routinely discarded following surgery, as a valuable source of EVs for myocardial infarction therapy. We also identify miR-1260a as protective of CM hypoxia.

**Supplementary Information:**

The online version contains supplementary material available at 10.1186/s13287-024-03983-y.

## Introduction

Preventing cardiomyocyte (CM) death during myocardial infarction (MI) and reperfusion injury is a desirable treatment goal, since they cannot be replaced by regeneration. Many approaches have been investigated to improve CM survival following hypoxia, including small molecules, biomaterials, cell therapy and cell products such as growth factors, cytokines, conditioned medium, and extracellular vesicles (EVs) [1–3]. Our research group has explored therapeutic use of stromal cells (CMSCLCs) derived from the human right atrial appendage (RAA) [4, 5]. In a previous study, less than 5% of intramyocardial injected CMSCLCs survived 24 h in the post-MI mouse heart, producing no therapeutic benefits. However, when CMSCLCs were encapsulated in a porous biomaterial to extend their survival, their secretome preserved ejection fraction and ventricular contractile parameters post-MI [6]. This adds to a growing body of evidence demonstrating that cell paracrine growth factors, cytokines and nucleic acids are important drivers of cardioprotection [7, 8]. Some pre-clinical investigations have shown EVs to be similar to superior to cell therapy post-MI, or that EVs can facilitate and improve cell therapy [9–11]. EVs from many sources including blood (plasma, serum or platelet EVs) and cultured cells, including bone marrow/amniotic/adipose/umbilical mesenchymal stromal cells (MSCs), pluripotent cells, cardiac progenitors, and cardiosphere-derived cells have found to have cardioprotective properties [1, 7, 10, 12–14]. However, reported mechanisms of EV therapeutic activity vary greatly between studies, ranging from transfer of specific miRNAs into CMs, binding of EV surface proteins to CM surface proteins (such as HSP70 binding to TLR4), enzymatic support of ATP generation, or direct transfer of intact mitochondria (by larger EVs) [15–19]. This heterogeneity is likely in part due to natural biological variability in EV phenotype and cargo, as well as methodological variability. For example, cardiac progenitor cells secrete multiple sub-populations of EVs which can each have specific activities [20]. A single population of EVs contains hundreds of miRNAs and proteins, each capable of influencing multiple pathways to bring about their effects [21]. EV cargo and function are strongly influenced by the type, origin and culture conditions of the donor cells [22]. For example, EVs from post-MI mouse hearts or activated macrophages worsen MI outcomes [23, 24]. EV miRNA cargo appears to be particularly important; studies have identified miR-21-5p, miR-125b, miR-30d, miR-486, miR-182 and miR-210 as important in post-MI responses [25–29]. These miRNAs are found in EVs of many origins and have multiple functions, including steering post-injury inflammation, promoting angiogenesis, protecting mitochondrial function, reducing myofibroblast-driven remodelling, modulating CM apoptosis, autophagy, and others.

Our previous work found that the CMSCLC secretome was protective post-MI in mice, but we did not characterise EVs or test them using human model systems. Therefore, this study aims to characterise their biophysical properties, miRNA cargo, and ability to protect hypoxic human CMs. Methodological variations in EV isolation, characterisation and assessments of functional activity make it challenging to compare different EV types across different studies [22]. To address this, we compared CMSCLC EVs to bone marrow MSC-EVs (BM-MSC-EVs, B_EVs), using standardised methods and reporting all parameters in line with best practice guidelines [22]. BM-MSCs were chosen for comparison since they are well-characterised and already have been used in human clinical trials of post-MI therapy, with predominantly positive results [30]. BM-MSC EVs specifically have been shown to significantly reduce cardiac fibrosis and preserve ejection fraction % following MI in rodents, with miR-125b and miR-21 singled out as particularly influential in manifesting those benefits [18, 31]. Since EVs are secreted to fulfil specific biological functions, we hypothesised that CMSCLC EVs would offer superior protection of cardiomyocytes due to their own cardiac origins. While many types of EVs have shown cardioprotective properties, the direct comparison of CMSCLC-EVs and BM-MSC EVs in this study allows an opportunity to identify EV populations that are most effective in mitigating hypoxia-driven damage in cardiomyocytes. Since the RAA is removed during cardiac surgeries, this research may provide new possibilities for therapeutic cells and EVs. As both EV populations originate from humans and carry human cargo, we used human induced pluripotent cell-derived cardiomyocytes (hiPSC-CMs) as our testing platform to increase translational relevance. To induce injury simulating MI we followed a well-validated hypoxia and nutrient deprivation injury model [20, 32]. Studies have shown benefits of EVs in pre-clinical animal models and small-scale, early-stage clinical trials, as recently reviewed [33]. However, more research is needed to better understand the interactions of EVs with injured human cells and to compare the efficacy of EVs from different sources.

## Methods

### Cell isolation and culture

Human subjects undergoing cardiac surgery were recruited by Dr. Chuan-Chih Hsu at Taipei Medical University Hospital. Human sample collection was carried out with ethical approval and supervision from Taipei Medical University IRB, protocol number N201910027, with David J. Lundy and Chuan-Chih Hsu as the principal investigators. All donors gave informed consent for tissue donation. Anonymised donor age, sex, and the specific usage of each donor-derived cell line are displayed in Table 1. In this study we excluded patients with significant confounding comorbidities such as cancer, chemotherapy, diabetes mellitus or significant renal disease. CMSCLCs were isolated and cultured as previously described [4, 6]. The RAA was collected and transported in 4˚C serum-free alpha-MEM (Thermo, 12571063) with 100 IU/ml penicillin and 100 µg/ml streptomycin (P/S), washed three times with PBS. The sample was trimmed to remove external fat and/or obvious scar tissue, cut into 2–4 mm pieces and digested with Pronase (0.01% w/v) at 37 ˚C for 45 min with agitation. The sample was then dissociated using a MACS Gentle Dissociator and filtered through a 70 μm cell strainer. The suspension was then centrifuged and suspended in 6 ml fresh alpha-MEM with 10% (v/v) foetal bovine serum (FBS) (Hyclone, SH30396.03) and 5 ng/ml recombinant human FGF2 (Peprotech 100–18 C), then seeded into a T25 flask. Cultures were routinely maintained at 5% CO_2_ 90% N_2_. Medium was replaced at day three and day seven, and colonies were counted at day seven. CMSCLCs were first passaged when approximately 90% confluent, which was typically day 10 to fourteen. Human induced pluripotent stem cell-derived cardiomyocytes (hiPSC-CMs), differentiated from healthy donor peripheral blood mononuclear cells (PBMCs), were purchased from the Taiwan Human Disease iPS Cell Service Consortium core facility, Academia Sinica, Taiwan. hiPSC-CMs were ≥ 90% cardiac troponin I positive by flow cytometry and were seeded at 180,000 cells/cm^2^ on growth factor-reduced Matrigel (Corning, 354230)-coated dishes (1:200) in RPMI 1640 (Gibco, 11875093) with B-27 (Gibco, 17504044) and 10 µM Y-27,632 ROCK inhibitor cocktail (Selleckchem, s1049). After two days, medium was changed to RPMI with B27 and changed every other day thereafter. TrypLE (Gibco, 12605028) reagent was used for re-plating. hiPSC-CMs were maintained for a minimum of 14 days before experiments were carried out. BM-MSCs from three healthy human donors (38157, 39060, 39334) were purchased from Lonza (PT-2501) and cultured in MSCGM with supplements (PT-3238 and PT-3011). All CMSCLCs and BM-MSCs described in this manuscript were between passages two to four, unless specifically stated otherwise.

## Cell differentiation

CMSCLC and BM-MSC differentiation was induced using StemPro^®^ differentiation kits (Thermo A1007201, A1007101 and A1007001) in 6-well plates following the manufacturer protocols. Differentiated cultures were stained with Oil Red O, Safranin O or Alizarin Red and quantified by dissolving in DMSO and reading absorbance at 520, 530 and 515 nm respectively. Gene expression was also used to assess cell differentiation using genes outlined in Supplemental Table 1.

## Cell hypoxia and cell viability assays

Human iPSC-CMs were subjected to normoxia (5% CO_2_ incubator) or 48 h hypoxia (Anaeropack, Mitsubishi), which is a validated injury model [20, 32, 34]. iPSC-CMs were incubated with basal, serum-free culture medium supplemented with equal volumes (2% v/v) of vehicle control (0.2 μm-filtered PBS) or EVs. B_EVs and C_EVs were normalised by both particle count and protein concentration, provided at 67 ng EV protein/µl culture medium, equivalent to approximately 2,000 EVs per hiPSC-CM. Hypoxia medium was pre-incubated under anaerobic conditions for at least 90 min before addition to cells. Cell membrane disruption and cytoplasmic leakage were measured using LDH assay (Dojindo, CK12-20) as a sensitive method to detect cardiomyocyte injury [35, 36]. Fresh medium (without EVs) was then added containing 10% (v/v) CCK-8 reaget (Boster, AR1160) and incubated for four hours to measure cellular metabolic activity. EVs were confirmed to have no effect on LDH or CCK-8 results compared to basal medium. Rat cardiac H9C2 cells (Taiwan BCRC, 60096) and human cardiomyocytes (AC16 Merck, SCC109) were used for some supporting experiments and their viability was measured by CCK-8 and LDH assays by the same methods.

## Flow cytometry

CMSCLCs or BM-MSCs were harvested, counted, and washed with staining buffer (PBS, with 2% v/v FBS). Cells were simultaneously stained by anti-human CD19-FITC (BD, 555412), CD44-PE (BD, 555479), CD45-PerCP-Cy5.5 (BD, 564105), CD105-APC (BD, 562408), CD166-BV421 (BD, 562936) antibodies for 10 min at room temperature in the dark. Cells were characterised using a BD FACSCanto II flow analyser. Isotype controls were evaluated to confirm specific binding of the antibodies against each target site. Single colour controls were used for each dye and compensation was applied. Unstained CMSCLCs (autofluorescence control) were used for gating. All cells above the signal threshold of the negative population were considered positive. Peripheral blood mononuclear cells (PBMCs) isolated by Ficoll tube were used as a positive control for CD19 and CD45.

## Semi-quantitative reverse transcription PCR

CMSCLCs and BM-MSCs were lysed with Trizol reagent and RNA was extracted using isolation columns (Qiagen 74104 or 74106). Reverse transcription was carried out using SuperScript IV (Thermo, 18-091-050) in a Thermo StepOne Plus thermocycler. Amplification used SYBR Green (Thermo, 43-687-08). Primers (shown in **Supplementary Table 1**) were used to detect gene expression which was normalised to GAPDH, unless otherwise stated. Each primer pair was tested without cDNA and confirmed to lack amplification.

### Extracellular vesicle isolation and characterisation

For EV harvesting, CMSCLCs and BM-MSCs were cultured in medium containing commercial EV-depleted FBS (Thermo, A2720801) to minimise interference from bovine EVs in downstream analyses. We have previously shown that EV content in this product was reduced by > 99% compared to standard FBS [6]. CMSCLCs and BM-MSCs were seeded at the same density (4,000 cells/cm^2^) in 15 cm dishes and conditioned medium was collected after 3 days from cells between 70 and 80% confluence. Unconditioned basal medium was used as a control in some experiments. Medium was first centrifuged at 500 g for 10 min at 4˚C, then 3,000 g for 20 min at 4˚C, then filtered through a 0.22 μm filter. EVs were isolated by ultracentrifugation of conditioned medium at 100,000 g for 16 h at 4˚C. The pellet was resuspended in 0.22 μm-filtered PBS and centrifuged again at 100,000 g for 70 min at 4˚C. The final pellet was resuspended in 0.22 μm-filtered PBS and stored in single-use aliquots at -80˚C until use. EVs were characterised in line with key MISEV23 recommendations using particle size, multiple surface and cargo protein markers, and morphological analyses [37]. Nanoparticle Tracking Analysis (NTA) (Malvern Nanosight NS-300) was used to measure particle concentration and size; samples were diluted to between 50 and 200 particles per frame. Conventional TEM (Hitachi HT7700) and cryoEM were used to visualise the isolated EVs. For cryoEM, samples were suspended in 0.22 μm-filtered PBS, prepared by FEI VitroBot-2 then imaged using a Tecnai F20 operated by a core facility technician. Lastly, EV proteins were analysed using ExoCheck (System Bio, EXORAY210B) antibody array membranes for human protein surface markers, cargo markers, and positive/negative controls. 50 µg total protein was added to the array, which was developed following the manufacturer instructions. The arrays were imaged using an iBright CL650 system. For Western blot, equal protein concentrations of EVs or whole-cell lysates were run on pre-cast gels (Biorad, 4561034), transferred to PVDF membranes, and detected using human-specific antibodies against CD9 and HSP70 (SystemBio, ExoAb) or GAPDH (Genetex, GTX100118) followed by goat anti-rabbit HRP antibodies.

## Apoptosis and cytokine antibody arrays

Human-specific antibody-based membrane arrays (AbCam, ab133998) were used to detect secreted proteins. Conditioned medium was collected, centrifuged to concentrate EVs, filtered to remove debris, then mixed 1:1 with lysis buffer to release EV-bound peptides. The array membrane was blocked using the provided buffer, then 1 ml of sample was added and incubated overnight at 4 ˚C with a gentle rocking motion. The remaining washing, biotin, HRP-streptavidin and development steps were performed according to the manufacturer protocol. Images were captured (iBright CL650) at an exposure level where no spots were fully saturated and the negative control area showed no signal. ImageJ was used to measure the integrated density of each spot. Complete basal culture media (without cell conditioning) was used as a blank sample to account for non-specific binding. Values for each cytokine from the blank sample were then subtracted from the conditioned medium samples. The membranes were normalised to one another using inbuilt positive controls on each membrane. A small amount of TIMP-1 background was noted in the blank. The basal culture medium contains rhFGF2, which was not included in the array. There was no noticeable crossover to other FGF family antibodies present on the array. For apoptosis analysis Abcam ab134001 was used. hiPSC-CMs from each group were lysed in the provided lysis buffer (with protease inhibitor cocktail), quantified by BCA, then diluted in deionised H_2_O to within assay range. 45 µg total protein was added, then incubated for 19 h, followed by biotin-cytokine incubation for 21 h and HRP-streptavidin for 24 h. Each washing step followed the manufacturer protocol with adjusted optional large volume washing steps using 6 ml wash buffer for 30 min. For detection, the manufacturer protocol was then followed, and images were captured and quantified as described above.

## EV miRNA arrays

miRNAs were extracted from EVs isolated from three separate donor lines per cell type using miRNeasy kits (Qiagen, 217684 or 217084). Spike-in miRNAs (Qiagen, 339390) were used to verify successful miRNA isolation, reverse transcription and amplification. Quality control plates (Qiagen, YAHS-999YC-2) were run for each preparation. Following successful QC, a 384-well format qPCR-based array (Qiagen, 339322, YAHS-312YG-8, V5) was used to detect 752 known miRNAs using a Roche LightCycler 480 analyser. Reverse transcription used a LNA RT kit (Qiagen, 339340) and amplification used an LNA SYBR green PCR kit (Qiagen, 339347), following manufacturer protocols. Cycle threshold (CT) values were determined, and plates were normalised to one another using built-in inter-plate calibration (IPC) wells, which were within ≤ 0.5 CT between all plates. A blank sample (water) was present in each plate and was confirmed to be negative. Spike-in samples (UniSP 2, 3, 4, 5) were consistent across all plates to within < 1 CT. miRNA threshold values were then normalised to a combination of geNorm-determined reference miRNAs using Qiagen GeneGlobe [38]. miR-16-5p, which has been previously shown as a suitable reference gene for cardiac tissues, was also included for normalisation [39]. Samples with CT values of ≥ 36.00 were considered as very low expression. The DAVID functional annotation tool was used to predict target genes, biological pathway (BP) and cellular component (CC) gene ontologies for the 50 most abundant miRNAs. Only targets present in both miRTarBase and Targetscan databases were included in our analyses. While searching, all synonyms of gene names were checked against NCBI databases to prevent loss of target genes.

### RNA-sequencing

RNA was extracted from hiPSC-CMs using a kit (Qiagen, 217084). Three repeats of each condition were performed across three separate experiments. RNA quality was assessed by NanoDrop, with 260/280 nm > 1.9 for all samples. Further QC was performed using an Agilent Bioanalyzer 2100 with RNA 6000 LabChip kit. RNA sample preparation was then carried out according to the Illumina protocol. The library was constructed from a SureSelect XT HS2 mRNA library preparation kit (Agilent, G999) and AMPure XP beads (Beckman Coulter) were used for size selection. Sequences were determined using Illumina sequencing-by-synthesis (SBS). Sequencing data (FASTQ reads) were generated using the Welgene Biotech pipeline based on bcl2fastq v2.20. StringTie v2.1.4 and DEseq v1.39.0 or DEseq2 v1.28.1 were used to perform differential expression analysis with genome bias detection/correction using blind mode. Functional enrichment assay in differentially expressed genes of each experiment design was performed using clusterProfiler v3.6. P-values were adjusted for false discovery rates using the Benjamini–Hochberg procedure.

### miRNA mimic transfection

miRNA mimics were purchased from Qiagen: miR-202-5p (GeneGlobe ID YM00472748), miR-1260a (YM00472820), miR-451a (YM00471387), miR-142-3p (YM00470805), miR-21-5p (YM00473093), negative control (YM00479902) and transfected into AC16 cells using 6 nl/µl HiPerFect transfection reagent. Mimics were provided at total concentrations of 5, 15 or 25 nM during hypoxia.

### Software, Statistics and Data Handling

FlowJo 10 was used to perform compensation, set gates, and quantify positive/negative cell populations for flow cytometry immunophenotyping. Microsoft Excel or Apple Numbers was used for data collection and calculations, including deltaCT values, qPCR normalisation, and calculating fold-changes. For RNA-seq analyses, a custom Java program was written to perform gene selection, synonym searching and subset analysis, based on RefSeq. Statistical analysis was performed using a custom Python script using publicly available scipy, matplotlib, bioinfokit and numpy packages. GraphPad Prism 10.2.2 (Mac) was used for statistical analysis and to generate graphs for publication. Individual data points are shown on graphs where possible. Sample sizes and statistical tests applied are described in the relevant figure legends and/or the text. The exact donor cells used for each experiment are shown in Table 1. ImageJ was used for calculating spot intensity of antibody membrane arrays and measuring EV sizes in cryoEM images. Final figures were assembled in Affinity Designer 1.10.6 and Apple Keynote 12.2.1.

## Results

### Cell characterisation

CMSCLCs were isolated from right atrial appendage (RAA) tissues obtained from patients undergoing coronary artery bypass grafting (CABG). Donor properties and cell uses are listed in Table 1:

**Table 1:** Properties of CMSCLC donors and use of cells

Donor	Age	Sex	Surgery/tissue	Notes	Cells used for
A	73	F	CABG/RAA	-	Immunophenotype Differentiation Cytokine array
B	67	M	CABG/RAA	-	Immunophenotype Differentiation Cytokine array EV miRNA
C	52	M	CABG/RAA	Triple vessel disease	Immunophenotype Differentiation Cytokine array
D	63	F	CABG/RAA	Aortic dissection.	EV miRNA hiPSC-CM protection hiPSC-CM RNA-seq
E	53	M	CABG/RAA	-	EV markers hiPSC-CM protection
F	58	M	CABG/RAA	-	EV miRNA

F = female, M = male, CABG = coronary artery bypass graft, RAA = right atrial appendage, EV = extracellular vesicle. hiPSC-CM = human induced pluripotent stem cell-derived cardiomyocyte

**Fig. 1:**
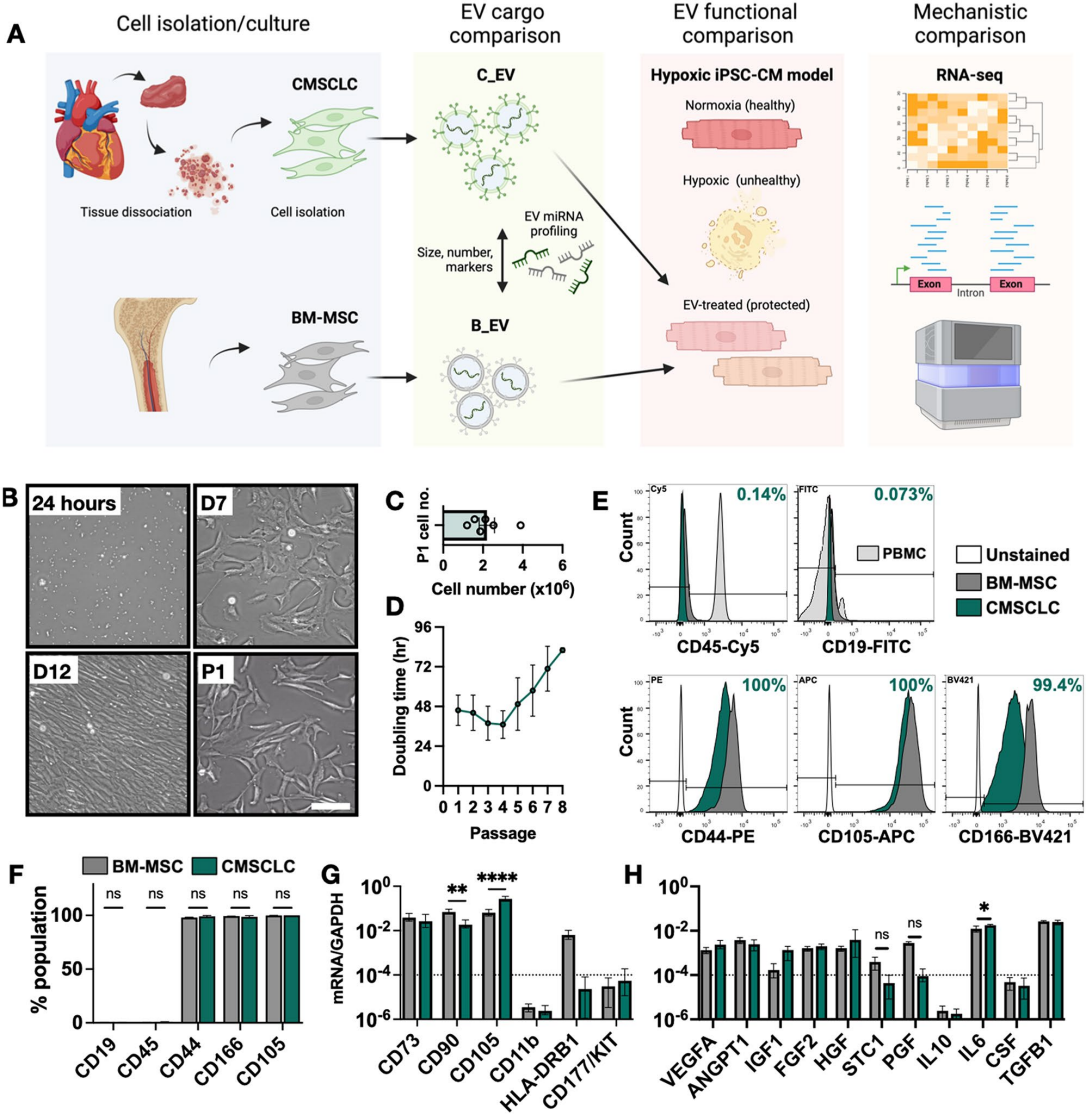
Comparison of cardiac and bone marrow-derived cells. (**A**) Schematic diagram of experimental design. (**B**) Images of CMSCLC morphology at 24 h, 7 days and 12 days after isolation, and 24 h after the first passage (P1). Scale bar 100 μm. (**C**) Number of viable cells obtained at passage 1 for *n* = 6 donor lines used in this study. (**D**) Cell doubling time plotted against passage number (average of *n* = 6 donors). (**E**) Representative histogram plots of flow cytometric analysis of CMSCLCs (green), BM-MSCs (dark grey) and unstained CMSCLCs (white). Negative markers CD19 and CD45, and positive markers CD44, CD105 and CD166 are shown. PBMCs (light grey) were used as positive controls for CD19 and CD45. (**F**) Quantification of flow cytometric analysis. *n* = 3 donors were compared to BM-MSCs by one-way ANOVA. (**G**) BM-MSC and CMSCLC (*n* = 3 donors) gene expression levels (as mRNA/GAPDH ratio) of positive and negative MSC markers. A dotted line shows the cut-off for low-expressed genes, which were considered negative. BM-MSC and CMSCLC samples were compared by two-way ANOVA with Sidak’s multiple comparison test. (**H**) BM-MSC and CMSCLC (*n* = 3 donors) gene expression of common MSC-associated paracrine factors. A dotted line shows the cut-off for low-expressed markers, which were considered negative. BM-MSC and CMSCLC samples were compared by two-way ANOVA with Sidak’s multiple comparison test. ns = not significant, ** = *P* ≤ 0.01, **** = *P* ≤ 0.0001. Pairs without annotations are also not significant (*P* > 0.05)

A schematic diagram of the overall experimental design is shown in Fig. 1A. Representative images showing the morphology of CMSCLCs at 24 h, 7 days, 12 days, and after the first passage (P1), are shown in Fig. 1B. At D7 colonies were visible and after D12 cells took on a spindle-shaped, fibroblastic morphology. At passage 1 an average of 2.20 × 10^6^ cells were obtained per donor (Fig. 1C). The mean cell doubling time (Fig. 1D), was ~ 40 h until after the fourth passage. CMSCLCs grew poorly in DMEM and were dependent on FGF2 (Supplementary Fig. 1). Flow cytometry was used to immunophenotype three separate donor lines, as shown in Fig. 1E, F. BM-MSCs and peripheral blood mononuclear cells (PBMCs) were used as positive controls. CMSCLC isolates at passage two were positive (≥ 99%) for CD44, CD105 and CD166 and negative (< 0.2%) for CD19 and CD45, as were all BM-MSCs. PBMCs were > 95% positive for CD45 and a subpopulation of PBMCs (approximately 3%) were positive for CD19, as expected. Gene expression levels of additional MSC markers are shown in Fig. 1G. CMSCLCs and BM-MSCs both met positive and negative ISCT-defined minimum criteria for MSCs [40]. We next analysed the capacity of these cells to carry out trilineage differentiation (Supplementary Fig. 2). CMSCLCs showed intracellular Oil Red O-positive droplets and calcified extracellular matrix after adipogenic and osteogenic differentiation, but BM-MSCs displayed significantly more. CMSCLCs showed little capacity for chondrogenesis. These observations were also reflected in expression of genetic markers pre/post differentiation.

### Examination of CMSCLC paracrine factors

Comparing gene expression levels of several well-known cardioprotective factors (Fig. 1H) showed that CMSCLCs expressed high levels of VEGFA, ANGPT1, IGF1, FGF2, HGF and TGFB1; equal to BM-MSCs from young healthy donors. We have validated in-house that CMSCLCs were negative for Islet-1 and NKX2.5, are NANOG positive, and express low levels of PDGFR-alpha. CMSCLCs also have low levels of p16 and SA-B-GAL at passage five [41]. Taking together CMSCLC surface markers, gene expression, colony formation ability, FGF2 dependence, and differentiation capacity these cells can be appropriately described as mesenchymal stromal cells [40]. 

Next, we tested whether EVs or other freely-secreted compounds were the most cardioprotective components of the CMSCLC secretome using hypoxic rat cardiomyoblast cells as a screening tool. The results (Supplementary Fig. 3A) showed that CMSCLC-conditioned medium was protective compared to basal medium (68% viability vs. 51% viability, *P* = 0.038). After ultracentrifugation, conditioned medium particle count was reduced by 96.3% (Supplementary Fig. 3B) and protein content was reduced by 29.9%, indicating successful EV depletion. This EV-depleted conditioned medium lacked any significant protective effects (58% viability, *P* = 0.423), whereas basal medium supplemented with isolated CMSCLC EVs (equalised by protein concentration) significantly protected cell viability (85.9%, *P* = 0.002). This demonstrates that EVs are the main protective component of the EV secretome. To measure other factors, we used an antibody array to detect 80 secreted cytokines and growth factors. Results from three CMSCLC donors are shown in Supplementary Fig. 3C-D. Proteins detected in conditioned medium included IL-8, IL-6, MCP-1/CCL2, TIMP-1 and − 2, osteoprotegerin and GRO-alpha. Eotaxin, angiogenin, IL-10 and VEGF were present at moderate concentrations and GM-CSF, CCL8, IGF-1 and FGF9 were detected in lower amounts.

**Fig. 2:**
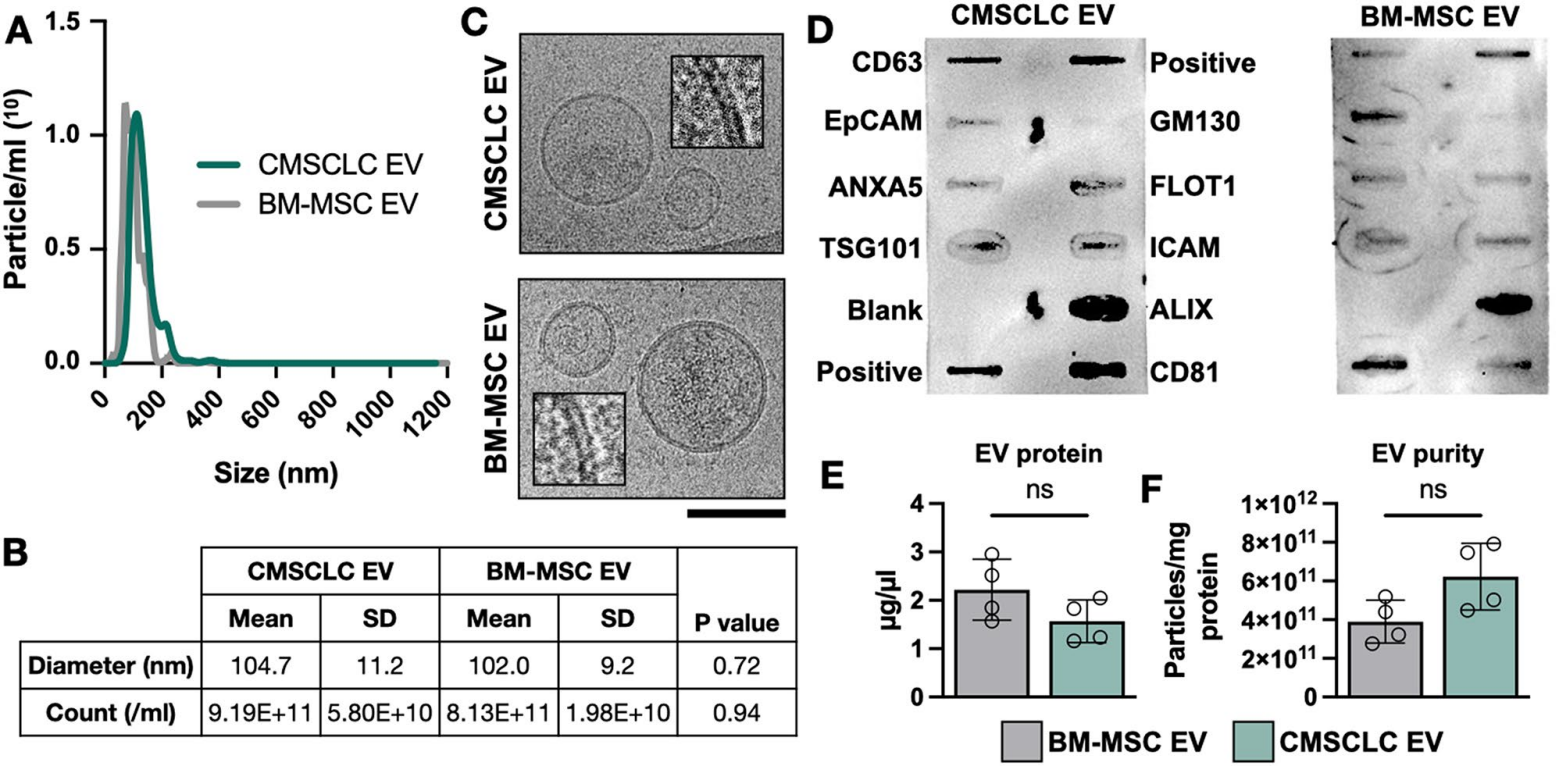
Extracellular vesicle isolation and characterisation. (**A**) Representative nanoparticle tracking analysis (NTA) size distribution plots for CMSCLC EVs (green) and BM-MSC EVs (grey). (**B**) Mean diameter and particle counts of *n* = 4 separate EV isolations and comparison by unpaired t-test. (**C**) Representative cryoEM images of isolated EVs. Scale bar 100 nm. A crop showing the lipid bilayer is also shown (inset). (**D**) Antibody-based membrane array showing human-specific EV surface markers and cargo markers. Two positive controls, a blank, and GM130 (cis-golgi marker) are also included. 50 µg total protein was added per membrane. (**E**) EV protein concentration (*n* = 4 per group) (**F**) Particle to protein ratio. Samples were compared by unpaired t-test. ns = not significant

### Extracellular vesicle isolation and characterisation

Next, we compared isolated CMSCLC EVs (C_EVs) and BM-MSC EVs (B_EVs). Nanoparticle tracking analysis (NTA) showed single peaks for both types of EV (Fig. 2A), as typical from ultracentrifugation. Over four separate batches, the mean particle size measured by NTA was 104.7 ± 11.2 nm and mean particle count was 9.19 × 10^11^ ± 5.80 × 10^10^ particles/ml for C_EVs, which was very similar to B_EVs (Fig. 2B). Conventional TEM (Supplementary Fig. 4A) showed “cup shaped” particles of approximately 100 nm diameter for both EV isolations. CryoEM (Fig. 2C) confirmed the presence of abundant spherical 50–200 nm diameter vesicles with lipid bilayer membranes in both isolates. Vesicle diameters were measured with an average diameter of 108.1 ± 4.2 nm for C_EVs and 107.3 ± 7.1 nm for B_EVs (Supplementary Fig. 4B), in close agreement with the NTA results (Fig. 2B). We then detected EV protein markers, as shown in Fig. 2D. Tetraspanin EV surface markers CD63 and CD81 were present in both populations, as were cargo markers ALIX, Flotillin 1, ICAM, TSG101 and ANXA5. GM130, a cis-golgi marker protein, showed only a faint signal, indicating low levels of contamination with non-EV cellular components. The EV protein concentration did not differ between four separate batches of C_EVs and B_EVs (Fig. 2E). Neither did the particle/protein ratio (Fig. 2F), which was in the range of 2–8 × 10^11^ particles/mg protein, indicating a high purity of EVs. Since we controlled cell density and standardised medium collection and EV isolation procedures, these results indicate that CMSCLCs and BM-MSCs produce EVs at a similar rate [22]. Lastly, we used Western blot to confirm additional EV markers for C_EVs (Supplementary Fig. 4C). HSP70 was detected in EVs and whole CMSCLC lysates (WCL), GAPDH was weaker in EVs compared to WCL while CD9 was enriched in EVs compared to WCL. These data show that CMSCLC- and BM-MSC-derived EVs were successfully isolated at high purity, suitable for further experimentation.

**Fig. 3:**
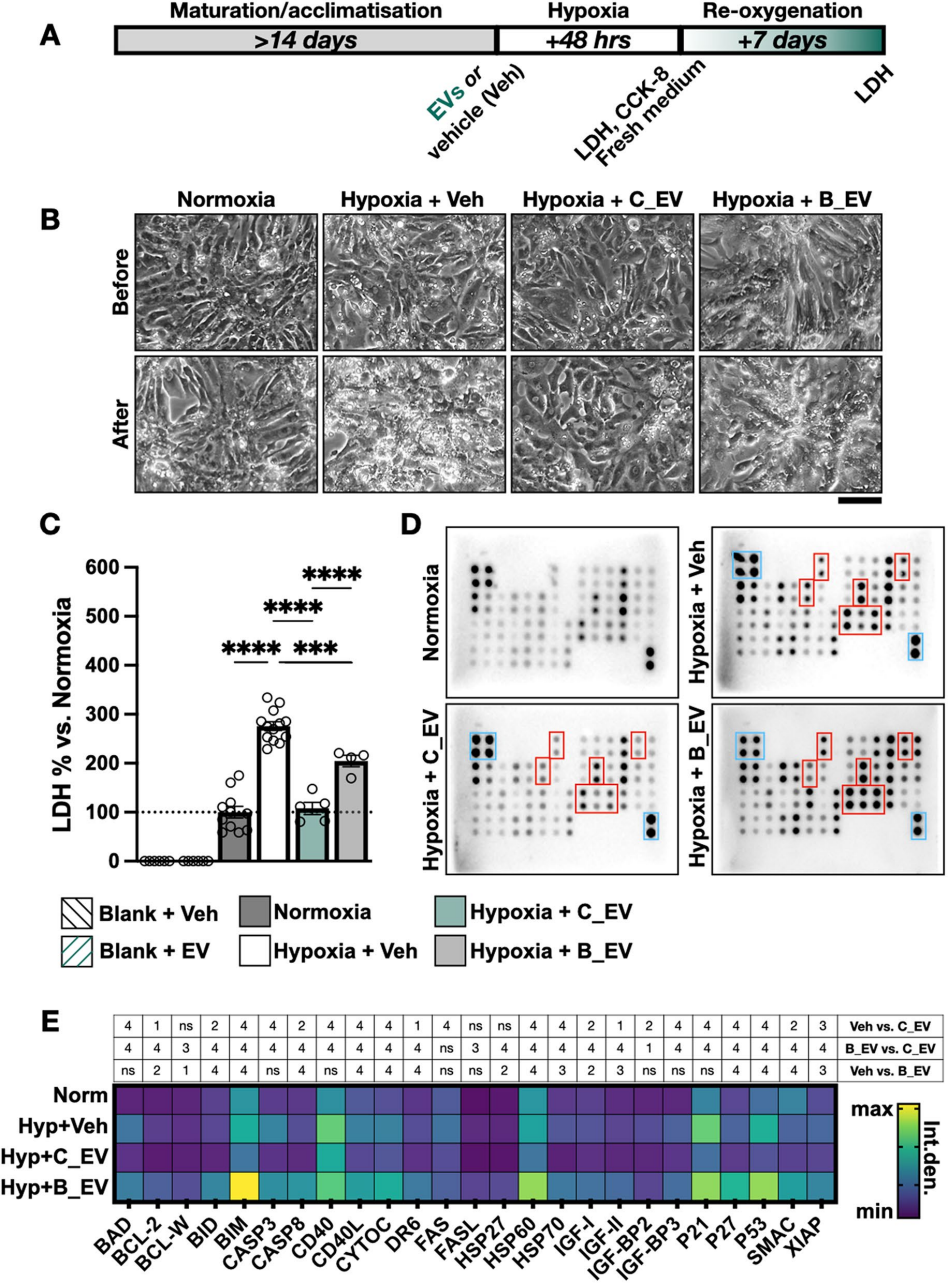
Protection of hypoxic human cardiomyocytes using CMSCLC and BM-MSC EVs (**A**) Experimental design showing hiPSC-CM seeding and hypoxia treatment. (**B**) Example images of hiPSC-CMs following 48 h normoxia or hypoxia + vehicle (Veh), or hypoxia with 67 ng/µl CMSCLC EVs (C_EVs) or BM-MSC EVs (B_EVs). Scale bar 100 μm. (**C**) Culture medium LDH levels after 48 h of hiPSC-CM exposure to each treatment group. Blank samples (without hiPSC-CMs) are also included. Hypoxic hiPSC-CM groups were compared by one-way ANOVA with Tukey’s multiple comparison test. *** = *P* ≤ 0.001, **** = *P* ≤ 0.0001 (**D**) Apoptosis protein arrays from each group (*n* = 2 per group). Examples of significant differences between samples are highlighted with red boxes. Positive controls are shown as blue boxes in the upper left and lower right corners. (**E**) Heatmap showing quantification of integrated density of high concentration apoptosis-related proteins (*n* = 2 per group). All groups were compared using two-way ANOVA with Tukey’s post-test. The table above the heat map describes statistical significance; 1 = *P* < 0.05, 2 = *P* ≤ 0.01, 3 = *P* ≤ 0.001, 4 = *P* ≤ 0.0001, ns = not significant (*P* > 0.05)

### Protection of hypoxic human cardiomyocytes using CMSCLC and BM-MSC EVs

Next, we compared the ability of C_EVs and B_EVs to protect hypoxic hiPSC-CMs. LDH release was used as a sensitive metric to measure hiPSC-CM damage at two time points [36]. The experimental design is shown in Fig. 3A. 48 h hypoxia was used as an injury model based on previous studies, resulting in 30–40% cardiomyocyte death [20, 32]. A time course of hypoxic injury is shown in Supplemental Fig. 5A. Hypoxic cells showed noticeable vacuolisation, with fragmentation and plentiful debris (Fig. 3B). B_EV-treated hiPSC-CMs had improved morphology, but C_EV-treated cells appeared more like normoxic cells. As expected, hypoxia resulted in significant (*P* ≤ 0.0001) LDH release compared to normoxia (Fig. 3C). Based on lysing hiPSC-CMs and measuring total LDH release, this corresponded to ~ 35% cell death. LDH release was completely prevented by C_EVs (1.08-fold, *P* = 0.99). hiPSC-CMs treated with B_EVs at the same dose had significantly lower LDH than the vehicle control (*P* = 0.0003), but they were not as effective as C_EVs. To detect hiPSC-CM apoptosis we used an antibody array to measure multiple apoptosis-related proteins (Fig. 3D, E). Hypoxia significantly increased pro-apoptotic protein expression, which were significantly lowered by C_EVs, including activated caspase 3 and 8, cytochrome C and p53. hiPSC-CMs treated with B_EVs had higher expression of most pro-apoptotic markers than C_EV-treated cells. Some additional experiments were conducted using C_EVs. Seven days after restoring hiPSC-CMs to normoxia (in fresh culture medium, without EVs) the control group showed further elevation of LDH release due to re-oxygenation injury (Supplementary Fig. 5B) [36]. However, hiPSC-CMs which were previously incubated with C_EVs showed significantly less LDH release (*P* = 0.03), eight days after the EV treatment ended, demonstrating that C_EVs had a long-lasting protective effect. A higher dose of C_EVs (167 ng/µl, ~ 5,000 EVs per cell) was also tested, which did not offer any benefit over 67 ng/µl (*P* = 0.68). Conventional viability assays (WST/CCK-8) found that hiPSC-CMs had very low baseline dehydrogenase activity under normoxic conditions, which increased during hypoxia (Supplementary Fig. 5C, D). H9C2 cells and AC16 cells both showed a large decrease in CCK-8 activity after hypoxia, while CMSCLCs were less affected. C_EVs increased CCK-8 conversion in a dose-dependent manner, implying that they may affect CM metabolism. Since cell-secreted EVs can contain cytoplasmic components such as LDH or dehydrogenases we confirmed (Fig. 3C and Supplementary Fig. 5C) that neither C_EVs nor B_EVs had any effect on the assays. Together, results show that C_EVs reduced membrane damage, apoptosis and cell death of hypoxic hiPSC-CMs more effectively than B_EVs.

### Extracellular vesicle miRNA cargo analysis

**Fig. 4:**
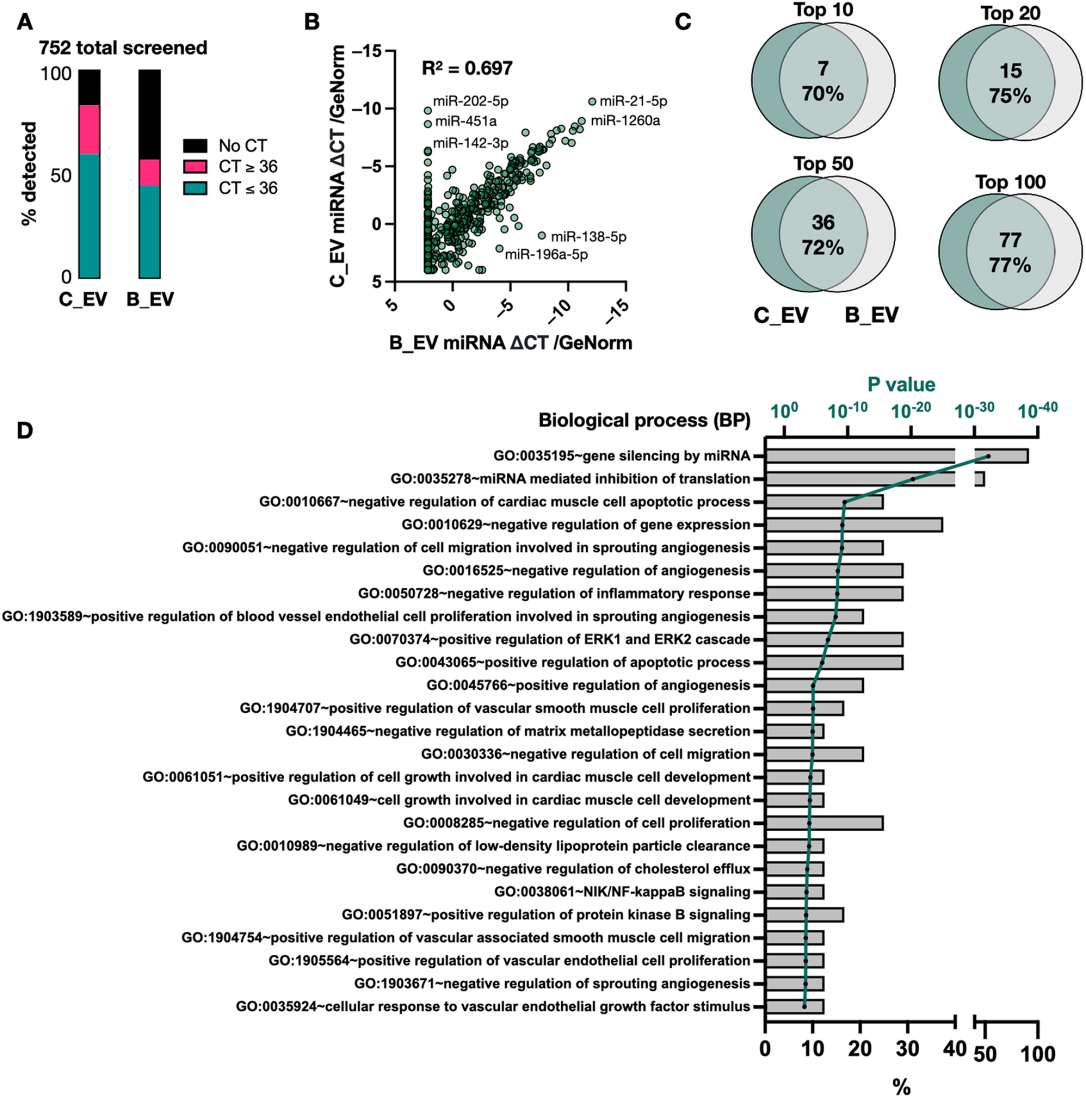
Extracellular vesicle miRNA cargo analysis. (**A**) Percentage of miRNAs detected in CMSCLC EVs (C_EV) and BM-MSC EVs (B_EVs) for three separate donor samples per group. Those with cycle threshold (CT) values of < 36.0 (green bar) were included in subsequent analyses. (**B**) Scatter plot of C_EV (Y axis) versus B_EV (X axis) mean miRNA expression levels normalised to reference miRNA (GeNorm) levels. The R-squared correlation is shown in the upper left. (**C**) Venn diagrams showing degree of overlap between the top 10, 20, 50 and 100 highest expressed C_EV miRNAs compared to B_EV miRNAs. (**D**) Gene ontology (GO) predictions for biological process (BP) for top 50 expressed C_EV miRNAs. Bars show the % of miRNAs belonging to each GO (lower X axis) and the green line shows the adjusted Fisher P value (upper X axis)

We next compared C_EV and B_EV miRNA cargo using three donors per cell type. Out of 752 probed miRNAs, 450 C_EV and 334 B_EV miRNAs were detected with cycle threshold (CT) values below 36 (Fig. 4A). All samples showed equal efficiency of miRNA isolation, reverse transcription and amplification (Supplementary Fig. 6A). Plotting normalised C_EV/ B_EV miRNA expression levels (Fig. 4B) showed a high correlation (R^2^ = 0.697). miR-21-5p was the highest detected miRNA in both EV types, and miR-1260a, miR-27a and miR-23a were highly-detected in both B_EVs and C_EVs. Comparing the most abundant miRNAs (Fig. 4C) showed 70–77% overlap between C_EV/B_EV cargo. Interestingly, some miRNAs (miR-202-5p (~ 11.6% of C_EV cargo), miR-451a (~ 5.1%) and miR-142-3p (~ 1.0%)) were found in high abundance in three separate C_EV donors but none of the B_EV samples. B_EVs contained hsa-miR-138-5p (1.0%) and hsa-miR-10b-5p (0.26%), which were not detected in C_EVs. Included among the most abundant miRNAs in both populations were miR-21-5p and miR-125b; both of which are stem cell-associated miRNAs [27]. 

### Target prediction of abundant CMSCLC and BM-MSC EV miRNAs

Since EV miRNAs act in combination to exert their effects, target pathway prediction was performed for the top 50 expressed C_EV and B_EV miRNAs. Categorisation by cellular component (CC) (Supplementary Fig. 6B) unsurprisingly showed high enrichment of exosome-related pathways. Categorisation by biological process (BP) (Fig. 4D) predicted GO:0010667 (negative regulation of cardiac muscle cell apoptotic process, (13 miRNAs, modified Fisher P-value = 1.6 × 10^− 8^)), angiogenesis (GO:1903589, GO:0016525), inflammation (GO:0050728), and cardiac muscle cell development (GO:0061049). The same analyses for B_EV miRNAs are shown in Supplementary Fig. 7. Due to the overlap between B_EV and C_EV miRNA cargo, target prediction results were overall similar.

### Transcriptomic analysis of CMSCLC and BM-MSC EV activity

**Fig. 5:**
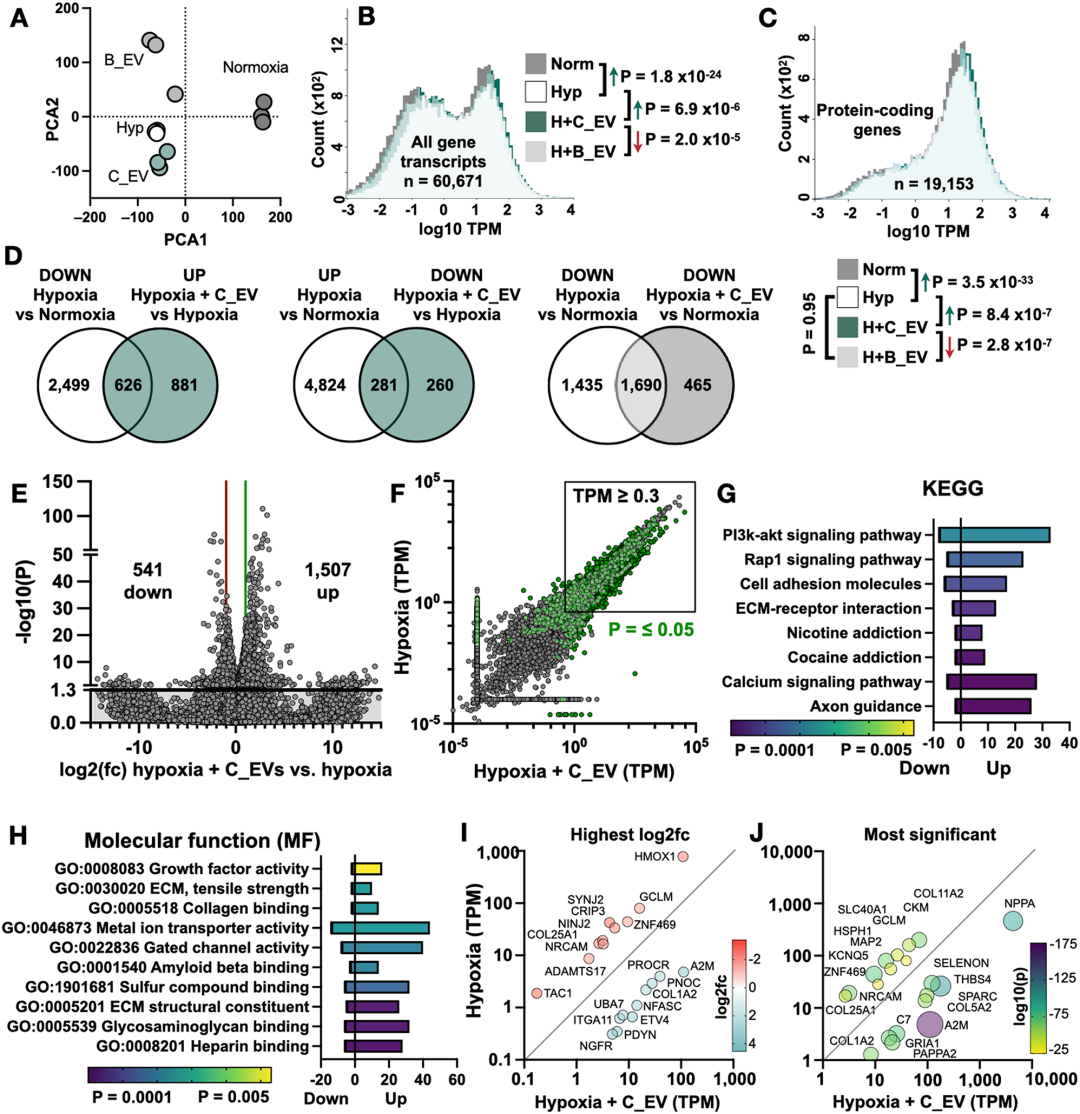
RNA sequencing of hypoxic EV-treated human cardiomyocytes. (**A**) Principal component analysis (PCA) for normoxia, hypoxia + vehicle (Hyp), hypoxia + CMSCLC EV (H + C_EV) and BM-MSC EV groups (H + B_EV). (**B**) TPM distribution of all gene transcripts or (**C**) protein-coding gene transcripts for the four experimental groups. Sample distributions were compared by Kolmogorov-Smirnov (KS) test, and the direction of change and P values are shown for each comparison. (**D**) Venn diagrams showing the number of overlapping genes between the stated comparisons. (**E**) Volcano plot of hypoxia + vehicle against hypoxia + C_EVs. The Y axis show statistical significance, with the solid line showing *P* = 0.05. The X axis shows log2 fold change with the red and green lines showing two-fold down- and up-regulation respectively. (**F**) Scatter plot of hypoxia + vehicle vs. hypoxia + C_EV. Each point represents one gene. Green points indicate *P* ≤ 0.05 and the box indicates the genes with ≥ 0.3 TPM which were included in subsequent analyses. (**G**) Pyramid plot of most significantly enriched pathways by KEGG for hypoxia + C_EV vs. hypoxia + vehicle. The X axis shows the number of upregulated and downregulated genes in each group and the bar colours indicate statistical significance. (**H**) Pyramid plot of molecular function (MF). (**I**) Scatter plot showing the 10 most differentially-expressed and most statistically significant genes (**J**) between hypoxia + vehicle vs. hypoxia + C_EV groups

Next, we used RNA-seq to examine how B_EVs or C_EVs affected the hypoxic hiPSC-CM transcriptome. 97.63 ± 0.27% of transcripts were successfully mapped. Normoxic hiPSC-CMs expressed TNNT2 (7,397 transcripts per million, TPM), as well as CM markers TBX5, HEY2, MYL2, ACTN1, IRX4, GJA1 (connexin-43) and ATPA2 (SERCA2) and had low CDK1 (8.6 TPM) and FGF8 (0.2 TPM), indicating a ventricular CM phenotype [42, 43]. Principal components analysis (PCA) (Fig. 5A) showed distinct profiles for each experimental group, with high consistencies of samples within each group.

Plotting TPM distribution (Fig. 5B) of all genes (*n* = 60,671 total) showed that hypoxia increased overall expression levels (*P* = 1.8 × 10^− 24^ vs. normoxia, measured by Kolmogorov-Smirnov (KS) Test). Interestingly, C_EVs further increased total expression levels (*P* = 6.9 × 10^− 6^ vs. hypoxia) whereas B_EVs had no effect on overall gene expression levels (*P* = 0.99). The same finding was observed for protein-coding genes (Fig. 5C). Individual comparisons are shown in Supplementary Fig. 8A, B. Comparing hypoxic to normoxic hiPSC-CMs (Supplementary Fig. 8C-F) revealed 3,125 and 5,105 significantly down and up-regulated genes respectively. Unsurprisingly, hypoxic cells showed enriched pathways related to cellular stress, apoptosis, oxidoreductase activity, dehydrogenase activity, electron transport chain and muscle contraction. Hypoxia-related genes such as VEGFA and ENO2 were upregulated up to 40-fold in all three hypoxia groups and were not affected by either of the EV treatments. These findings are very similar to previously published microarray and RNA-seq of hypoxic human cardiomyocytes [32, 42]. This demonstrates that the utilised hypoxia model induced the relevant and appropriate responsive pathways in iPSC-CMs.

Venn diagrams comparing overlapping up- and down-regulated genes between normoxia/hypoxia/C_EV treatment groups are shown in Fig. 5D. C_EVs reversed the direction of many gene expression changes which were induced by hypoxia, mostly by increasing their expression. Comparing hypoxic hiPSC-CMs + C_EVs against EV vehicle (Fig. 5E, F) showed that C_EVs significantly up-regulated 1,507 genes and significantly down-regulated 541 genes. Categorising differentially expressed genes by KEGG (Fig. 5G) revealed significant up-regulation of Pi3k-akt signalling, ECM-receptor interaction, cell adhesion and calcium signalling pathways, all of which are important modulators of CM survival [36]. Sorting by molecular function (Fig. 5H), the most significant changes related to up-regulation of heparin and glycosaminoglycan (GAG) binding, ECM structural constituents, and metal ion transporter activity.

Next, we looked at the most differentially expressed genes in the C_EV-treated hiPSC-CMs by both fold-change and statistical significance (Fig. 5I, J). Of the most significantly upregulated genes, many are known to be cardioprotective, including A2M, NPPA, SELENON and THBS4. The most upregulated gene, A2M (alpha-2-macroglobulin), is a powerful anti-inflammatory protein which inhibits multiple cytokines and cellular proteases, and activates cardioprotective ERK1/2, Akt and PI3-kinase pathways. It was up-regulated 24-fold by C_EVs but unchanged by B_EVs. SELENON, coding for selenoprotein N, protects cells from oxidative stress and maintains calcium homeostasis and contractile function during stress. It was down-regulated by hypoxia, increased 4-fold by C_EVs (*P* = 1.12 × 10^− 70^) but was unchanged by B_EVs (*P* = 0.42). THBS4 has been previously shown as cardioprotective and was increased 6.8-fold by C_EVs and unaffected by B_EVs [44]. Together, these data indicate that C_EVs induced multiple protective responses in hypoxic cardiomyocytes.

Looking at the genes most significantly reduced by C_EVs, CKM (creatine kinase, M-type) was reduced by C_EVs but was increased by B_EVs. HMOX1 (heme oxygenase-1) was expressed at very low levels (2.2 TPM) in normoxia and increased to 774.1 TPM in hypoxia, as expected. Both C_EVs (108.2 TPM, *P* = 7.3 × 10^− 22^) and B_EVs (93.0 TPM, *P* = 4.81 × 10^− 41^) lowered HMOX1 expression. Interestingly, both CKM and HMOX1 are hypoxia-inducible and have cardioprotective functions; HMOX1 by anti-oxidant activity and CKM by preserving cardiomyocyte ATP production [45, 46]. Taken together, these data indicate that C_EVs significantly aided in up-regulating multiple cardioprotective genes in response to hypoxic stress, and many of these changes were not found after treatment with B_EVs. However, the genes which were strongly downregulated by C_EVs were mostly also downregulated by B_EVs.

**Fig. 6:**
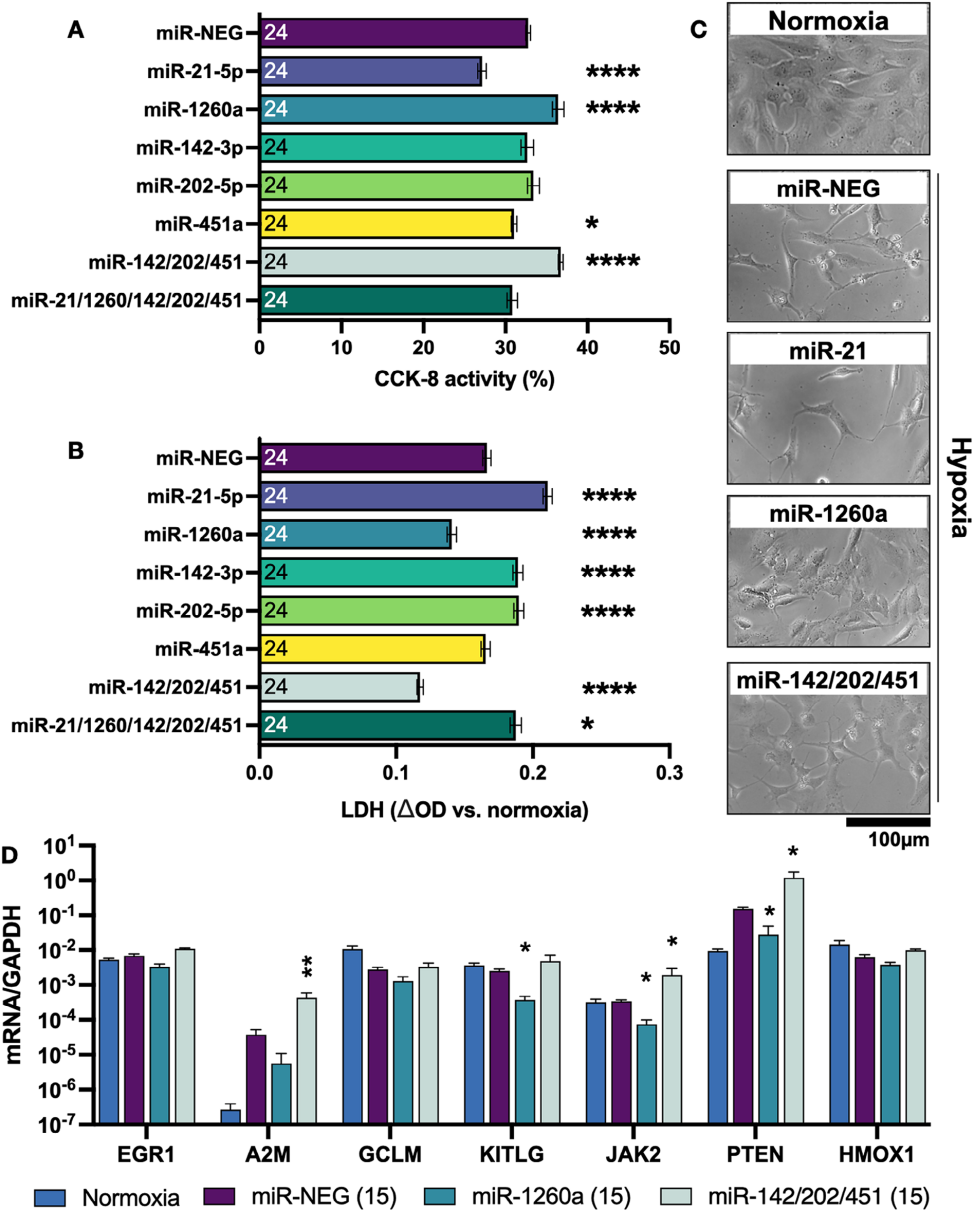
Determining hypoxia protection by abundant EV miRNAs. (**A**) CCK-8 activity of hypoxic AC16 cardiomyocytes incubated with miRNA negative control (miR-NEG), single miRNA mimics or combinations of mimics to a total of 15 nM. Percentages are relative to normoxia. (**B**) LDH secretion shown as change in absorbance relative to normoxia. Groups were compared to miR-NEG by one-way ANOVA with Dunnett’s multiple comparison test. * = *P* < 0.05, **** = *P* ≤ 0.0001. *N* = 24 samples per group. (**C**) Representative images from selected conditions. (**D**) Gene expression of predicted miRNA targets after normoxia, hypoxia + miR-NEG, miR-1260a or miR-142/202/451 at 15 nM. The Y axis shows log scale of gene expression normalised to GAPDH. *N* = 4 independent samples per bar. Statistical annotations show comparisons against hypoxia + miR-NEG by one-way ANOVA with Dunnett’s multiple comparison test. * = *P* < 0.05, ** = *P* ≤ 0.01

The hypoxic hiPSC-CM response to B_EVs is shown in Supplementary Fig. 9. Here, there was more total gene downregulation (1,719) than upregulation (1,407) and the most significantly enriched biological pathways related to ion channels, calcium signalling and cAMP signalling. B_EVs also affected many of the same pathways as C_EVs, including GO: 0008201 heparin binding (*P* = 0.02), and GO: 0005539 glycosaminoglycan binding (*P* = 0.011). Direct comparison of C_EVs and B_EVs is shown in Supplementary Fig. 10 and a summary of strongly differentially regulated genes (based on Fig. 6I-J) is shown in Supplementary Fig. 11A and CM apoptosis-related genes in Supplementary Fig. 11B.

### Integrating CMSCLC and BM-MSC EV miRNA cargo and down-regulated genes

Next, we integrated RNA-seq data with target prediction for the top 50 most abundant C_EV and B_EV miRNAs. We filtered for genes which were ≥ 2.0-fold up-regulated during hypoxia and ≥ 2.0-fold down-regulated with addition of C_EVs with p.adj < 0.05; nine genes met these criteria for C_EVs (Supplemental Fig. 12A). Of these 9 predicted genes, all except EGR1 were also significantly reduced by B_EVs. EGR1 was strongly up-regulated during hypoxia (6.1-fold), downregulated by C_EVs (4.1-fold, *P* = 0.001755), but not affected by B_EVs (*P* = 0.10). The same analysis was performed for B_EVs, as shown in Supplemental Fig. 6B.

To validate some of these findings we treated AC16 cardiomyocytes with miR-21-5p and miR-1260a, to represent the most abundant C_EV and B_EV miRNAs, as well as miR-202-5p, miR-451a and miR-142-3p to represent abundant C_EV-exclusive miRNAs. At a 5nM concentration (Supplemental Fig. 13a) miR-21-5p transfection reduced hypoxic CM viability compared to the negative control mimic, miR-1260a had no significant effect, and 5 nM of combined miR-142-3p/202-5p/451a increased viability. At 25 nM (Supplemental Fig. 13b), all miRNAs greatly and equally reduced cell viability, indicating saturation of the RISC system. At 15 nM (Fig. 6A) miR-21-5p again reduced viability, and miR-1260a increased viability compared to negative control transfection. Interestingly, single miR-142-3p, 202-5p or miR-451a at 15 nM had no significant effects on viability, but when combined (5 nM each), they significantly improved viability. A combination of the top 5 C_EV miRNAs (including miR-1260a and miR-21-5p) again had no significant effect, possibly from re-introduction of detrimental miR-21-5p. Measuring LDH release (Fig. 6B) showed the same findings of miR-1260a and miR-142/202/451 protecting hypoxic CMs, and miR-21 worsening CM injury. Representative images (Fig. 6C) also showed the detrimental effects of miR-21 and beneficial effects of miR-1260a and the C_EV-exclusive miR-142/202/451a combination. Gene expression analysis of the two cardioprotective miRNA treatments showed that some of the changes found in iPSC-CMs were also reproduced in CMs treated with miRNA mimics (Fig. 6D). For example, A2M increased with hypoxia, and was further increased by the combination of miR-142/202/451 (15.2-fold vs. miR-NEG). miR-1260a also reduced expression of several target genes including KITLG, JAK2 and PTEN. However, some of the C_EV modified targets such as HMOX1 and EGR1 did not change in response to these miRNA mimics, indicating that they may be targeted by other components of the EV cargo. Gene expression was also tested with mimics provided at 5 nM and 25 nM concentrations (Supplemental Fig. 13, d) which found similar trends. Notably, 5 nM of miR-142/202/451 was sufficient to increase A2M expression. Taken together, we summarise that EVs isolated from human RAA-derived cardiac stromal cells can robustly protect cardiomyocytes from hypoxic injury which is in part due to miRNA cargo and upregulating cardioprotective pathways.

## Discussion

Our results showed that EVs secreted by primary human RAA-derived stromal cells can protect CMs from hypoxic injury. A dose of C_EVs equivalent to ~ 2,000 EVs per recipient CM negated cell damage, reduced apoptosis, and provided long-lasting protection from reoxygenation damage in human iPSC-CMs. RNA-seq of iPSC-CMs showed that C_EVs induced activation of multiple known cardioprotective genes, particularly those related to handling oxidative stress. Some of these were recapitulated by abundant EV miRNAs alone. These data support results from a pilot study by our group which showed that transplanted encapsulated CMSCLCs had therapeutic effects in mouse MI and HLI models *via* their secretome [6]. It stands to reason that purified C_EVs would also have beneficial effects in animals; however, here we focused solely on protection of human CMs. hiPSC-CMs were used as the gold-standard in vitro model of human CMs [42]. 

Our study identified more than 300 miRNAs conserved in C_EVs and B_EVs from three different donors. To make our analyses more meaningful we focused on the top 50 most abundant miRNAs, which made up ~ 99% of the total miRNAs measured. miR-21-5p comprised ~ 20% of total C_EV and B_EV miRNA and has been previously associated with both MSCs and cardiac tissue; thus detecting it was not a surprise [18]. In MI, studies have shown beneficial effects of miR-21 delivery, and worse outcomes following miR-21 inhibition [28]. However, we found that miR-21-5p negatively affected viability of hypoxic human CMs. miR-125b was found in both C_EVs (8th most abundant) and B_EVs (3rd ). Members of our group have previously shown that endothelial cell-derived miR-125b can improve CM contractile properties, calcium handling, and maturation [47]. miR-125b has been shown to have multiple protective actions following MI. [27, 48] Another known cardioprotective miRNA, miR-19a, was also detected in both B_EVs and C_EVs [49]. Thus, it is likely that many of the protective benefits of C_EVs and B_EVs are derived through these known beneficial miRNAs.

In addition to these well-known miRNAs, we detected several miRNAs which are not well-described in the cardiac field. miR-1260a was the 3rd most abundant miRNA in C_EVs and 2nd in B_EVs, but there is little published information describing its function in CMs. One study observed miR-1260a up-regulation following MI surgery in sheep, but no mechanism was described [50]. Our data showed that miR-1260a alone can protect hypoxic human CMs; thus, some of the protective effects of C_EV and B_EV activity may also be ascribed to miR-1260a. miR-202-5p was the 2nd highest detected miRNA in three separate C_EV donors, comprising ~ 11.6% of total C_EV miRNA, but miR-202-5p was absent in all B_EV lines, suggesting a degree of cardiac specificity. The role of miR-202 in cardiac tissue is uncertain; both beneficial and detrimental effects have been shown in H9C2 and rat models [51, 52]. Our data showed that miR-202-5p alone had no significant effect on human CM injury during hypoxia. Despite relatively high similarity of C_EV and B_EV miRNA cargo, effects of each EV type on hypoxic hiPSC-CMs were clearly different. Therefore, we looked at three abundant C_EV-exclusive miRNAs; miR-451a, miR-142-3p and miR-202-5p. None of these miRNAs had significant effects on hypoxic CM viability when given alone, but they were protective when given as a combination, demonstrating that the EV cargo works in combination to bring about effects in target cells. RNA-seq of hypoxic EV-treated hiPSC-CMs showed that C_EVs strongly induced expression of several cardioprotective genes which were not induced by B_EVs. A2M was the most significantly increased gene by C_EVs, and this was also reproduced by addition of miR-451a/142/202. Direct injection of A2M in the post-I/R heart has been shown to prevent CM death and reduce infarct size [53]. C_EVs also strongly upregulated NPPA, coding for atrial natriuretic peptide (ANP), which reduced infarct size and improved cardiac ejection fraction in a human clinical trial; thus, its strong induction by C_EVs may serve a protective role [54]. Similarly, ERO1A (ER oxidation 1) was significantly increased by C_EVs. ERO1A enables oxidoreductase activity and protein re-folding, reducing apoptosis, preserves intracellular calcium homeostasis and provides a protective effect [55, 56]. 

Despite many pre-clinical studies, and a small number of early clinical trials, there are several barriers to translating EVs into clinical use [33]. There is variability between studies and labs in terms of isolating and characterising EVs, and considerably heterogeneity in EV population subtypes, even from the same cultured cells [20]. While EV miRNAs, proteins and lipids all contribute to their therapeutic effects, the importance and role of specific cargo remains unclear. By standardising isolation, cargo analysis and functional comparisons of EVs and cargo constituents, our work contributes to better understanding of EVs as a foundation for future clinical trials.

### Limitations

The present study has some limitations which should be considered. While we looked at miRNAs, they are not the only bioactive constituent of EVs. For example, EV-bound proteins such as HSPs and adiponectin can be cardioprotective [15, 57]. A similar study of cardiac progenitor cell EVs attributed cardioprotective effects to proteins such as PAPP-A and IGF-1 [58]. Additionally, EVs also contain bioactive metabolites, substrates and lipids; any of which may have influenced the hiPSC-CMs in our study. Indeed, not all C_EV effects were recapitulated by the most abundant miRNAs and we noted that the AC16 transcriptional responses to hypoxia were not identical to iPSC-CMs. Secondly, this study focused on the effects of B_EVs and C_EVs only on cardiomyocytes; but the overall response to MI in vivo involves other cell types such as cardiac fibroblasts, endothelial cells and macrophages. Thus, although we found that C_EVs offered better protection of CMs than B_EVs, it is possible that B_EVs may be superior to C_EVs in other aspects of the therapeutic response such as immunomodulation, angiogenesis or steering remodelling. This highlights the complexity of explaining EV therapeutic effects since they contain hundreds of miRNAs, each with hundreds of potential targets which may synergise or antagonise one another [21]. Additionally, while RNA-seq generates a comprehensive snapshot of gene expression, it does not confirm protein concentrations, function or activity. We also examined only one fixed time point following hypoxia and EV exposure, and should bear in mind that transcriptomic responses vary over time. Lastly, a comparison of B_EVs and C_EVs from the same donor could be a valuable addition; but obtaining bone marrow samples from the cardiac surgical patients was not within the scope of the ethical approvals for this project.

The RAA can be resected for cannula insertion during coronary artery bypass grafting (CABG), which is a commonly performed cardiac surgery [59]. Thus, this tissue could be a feasible source of therapeutic cells/EVs, in a similar manner to umbilical cord or adipose-derived MSCs which also originate from surgical waste.

## Conclusion

Here we describe the extracellular vesicles derived from human primary cardiac stromal cells (CMSCLCs). The CMSCLC EVs (C_EVs) had powerful protective effects on hypoxic human cardiomyocytes, reducing cell damage, apoptosis and death more effectively than BM-MSC EVs. Cargo profiling showed that C_EVs contained several well-known cardioprotective miRNAs, as well as some that are less well described, which we then demonstrated to have protective properties. Transcriptomic analysis revealed that C_EVs induced a robust up-regulation of several pro-survival pathways in hypoxic-injured cardiomyocytes, acting by different pathways than BM-MSC EVs. Many of the major changes were recapitulated by delivery of selected miRNAs from the C_EV cargo. We conclude that C_EVs are worthy of further investigation as a future treatment for myocardial infarction.

### Clinical perspectives

The study shows that extracellular vesicles from tissues routinely discarded following coronary artery bypass grafts have cardiomyocyte-protective potential equal or superior to known therapeutic MSCs. These cells could be translated in an autologous therapeutic setting, in a manner similar to adipose MSCs which are also derived from surgical wastes.

## Electronic supplementary material

Below is the link to the electronic supplementary material.

Supplementary Material 1

Supplementary Material 2

## Data Availability

RNA-seq data is available at EBI arrayexpress under accession number E-MTAB-13966 or NCBI at GSE275104. Other data can be made available upon reasonable request to the corresponding author.

## Abbreviations

BM: Bone marrow
CABG: Coronary artery bypass graft
CM: Cardiomyocyte
CMSCLC: Cardiac mesenchymal stromal cell-like cell
EV: Extracellular vesicle
H/R: Hypoxia/reoxygenation
iPSC-CM: Induced pluripotent stem cell-derived cardiomyocyte
LDH: Lactate dehydrogenase
MI: Myocardial infarction
MSC: Mesenchymal stromal cell
RAA: Right atrial appendage
