# Supplementary material for: Extracellular vesicles from human cardiac stromal cells up-regulate cardiomyocyte protective responses to hypoxia
Source: Stem Cell Res Ther. 2024 Oct 12;15:363. doi: 10.1186/s13287-024-03983-y (PMC11470622; doi:10.1186/s13287-024-03983-y)
Supplement: Supplementary file 1 — Supplementary Material 1 [file 13287_2024_3983_MOESM1_ESM.pdf]

Supplementary Information

Extracellular vesicles from human cardiac stromal cells up-regulate cardiomyocyte protective responses to hypoxia

Supplementary data

Supplementary Table 1: List of primers used in the study
Supplementary Figure 1: Additional CMSCLC growth characteristics
Supplementary Figure 2: CMSCLC differentiation analysis
Supplementary Figure 3: CMSCLC protein secretome data
Supplementary Figure 4: Additional CMSCLC EV characterisation
Supplementary Figure 5: Additional hiPSC-CM viability assays
Supplementary Figure 6: miRNA array quality control and additional pathway analysis
Supplementary Figure 7: miRNA target pathway prediction for BM-MSC EVs
Supplementary Figure 8: RNA-seq of hypoxia + vehicle vs. normoxia hiPSC-CMs
Supplementary Figure 9: RNA-seq of hypoxia + vehicle vs. BM-MSC EV hiPSC-CMs
Supplementary Figure 10: RNA-seq of hypoxia + CMSCLC EV vs. BM-MSC EV hiPSC-CMs
Supplementary Figure 11: Highly differentially expressed genes
Supplementary Figure 12: Multi-omic EV miRNA target prediction and RNA-seq integration
Supplementary Figure 13: 5 nM and 25 nM miRNA mimics and RT-qPCR

Gene	Forward	Reverse
	MSC markers	
CD73	AGTCCACTGGAGAGTTCCTGCA	TGAGAGGGTCATAACTGGGCAC
CD90	GAAGGTCCTCTACTTATCCGCC	TGATGCCCTCACACTTGACCAG
CD105	CGGTGGTCAATATCCTGTCGAG	AGGAAGTGTGGGCTGAGGTAGA
CD11B	GGAACGCCATTGTCTGCTTTCG	ATGCTGAGGTCATCCTGGCAGA
HLA-DRB1	GAGCAAGATGCTGAGTGGAGTC	CTGTTGGCTGAAGTCCAGAGTG
CD177/KIT	CAACCTGCTCAATGGGACACAG	CTTGAGCCAAGTTTCCGTGTGTC
	Paracrine factors	
VEGFA	TTGCCTTGCTGCTCTACCTCCA	GATGGCAGTAGCTGCGCTGATA
ANGPT1	CAACAGTGTCCTTCAGAAGCAGC	CCAGCTTGATATACATCTGCACAG
IGF1	CTCTTCAGTTCGTGTGTGGAGAC	CAGCCTCCTTAGATCACAGCTC
FGF2	AGCGGCTGTACTGCAAAAACGG	CCTTTGATAGACACAACTCCTCTC
HGF	GAGAGTTGGGTTCTTACTGCACG	CTCATCTCCTCTTCCGTGGACA
STC1	GCAGGAAGAGTGCTACAGCAAG	CATTCCAGCAGGCTTCGGACAA
PGF	GGCGATGAGAATCTGCACTGTG	ATTCGCAGCGAACGTGCTGAGA
IL10	TCTCCGAGATGCCTTCAGCAGA	TCAGACAAGGCTTGGCAACCCA
IL6	AGACAGCCACTCACCTCTTCAG	TTCTGCCAGTGCCTCTTTGCTG
CSF	TGAGACACCTCTCCAGTTGCTG	GCAATCAGGCTTGGTCACCACA
TGFB1	TACCTGAACCCGTGTTGCTCTC	GTTGCTGAGGTATCGCCAGGAA
	Adipogenesis markers	
LEP	GCTGTGCCCATCCAAAAAGTCC	CCCAGGAATGAAGTCCAAACCG
LPL	CTGCTGGCATTGCAGGAAGTCT	CATCAGGAGAAAGACGACTCGG
ADIPOQ	CAGGCCGTGATGGCAGAGATG	GGTTTCACCGATGTCTCCCTTAG
FABP4	ACGAGAGGATGATAAACTGGTGG	GCGAACTTCAGTCCAGGTCAAC
SLC27A1	TGACAGTCGTCCTCCGCAAGAA	CTTCAGCAGGTAGCGGCAGATC
	Osteogenesis markers	
ALPL	GCTGTAAGGACATCGCCTACCA	CCTGGCTTTCTCGTCACTCTCA
BGLAP	CGCTACCTGTATCAATGGCTGG	CTCCTGAAAGCCGATGTGGTCA
BSP	GGCAGTAGTGACTCATCCGAAG	GAAAGTGTGGTATTCTCAGCCTC
	Chondrogenesis markers	
OTOR	GCTGGTAAAAGAAAATGGAGCTGG	CACACGCTGTTCCTTGACCAAG
CHAD	CCTTTGGCAGATACCTGGAGAC	GCTGGTTCAAGCGGTTGTTCTC
COL10A1	CGCTGAACGATACCAAATGCCC	TGGACCAGGAGTACCTTGCTCT
COL2A1	CCTGGCAAAGATGGTGAGACAG	CCTGGTTTTCCACCTTCACCTG
miRNA targets and significant RNA-seq changes		
EGR1	AGCAGCACCTTCAACCCTCAGG	GAGTGGTTTGGCTGGGGTAACT
A2M	GTTGAAGAGCCTCACACGGAGA	TTCCACTCGGTGATGGTGTCAG
GCLM	TCTTGCCTCCTGCTGTGTGATG	TTGGAAACTTGCTTCAGAAAGCAG
KITLG	CTGGAGACTCCAGCCTACACTG	CTGCCCTTGTAAGACTTGGCTG
JAK2	CCAGATGGAAACTGTTCGCTCAG	GAGGTTGGTACATCAGAAACACC
PTEN	TGAGTTCCCTCAGCCGTTACCT	GAGGTTTCCTCTGGTCCTGGTA
HMOX1	CCAGGCAGAGAATGCTGAGTTC	AAGACTGGGCTCTCCTTGTTGC
	Housekeeping	
GAPDH	GTCTCCTCTGACTTCAACAGCG	ACCACCCTGTTGCTGTAGCCAA

Supplementary Table 1. List of primers used in the study. All primers are against human targets.

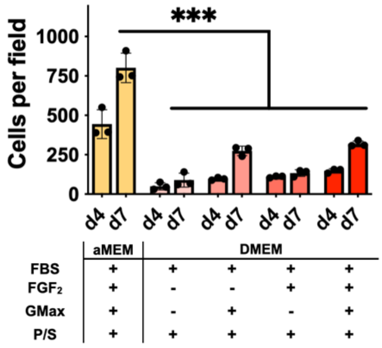

Supplementary Figure 1. Evaluation of cell growth (n = 1 donor line, 3 experimental replicates) based on cell number at 4 and 7 days after seeding into alpha-MEM or DMEM with or without FBS, FGF_2, Glutamate (GMax) and/or penicillin/streptomycin. Data were compared by two-way ANOVA. *** = $P \leq 0.001$

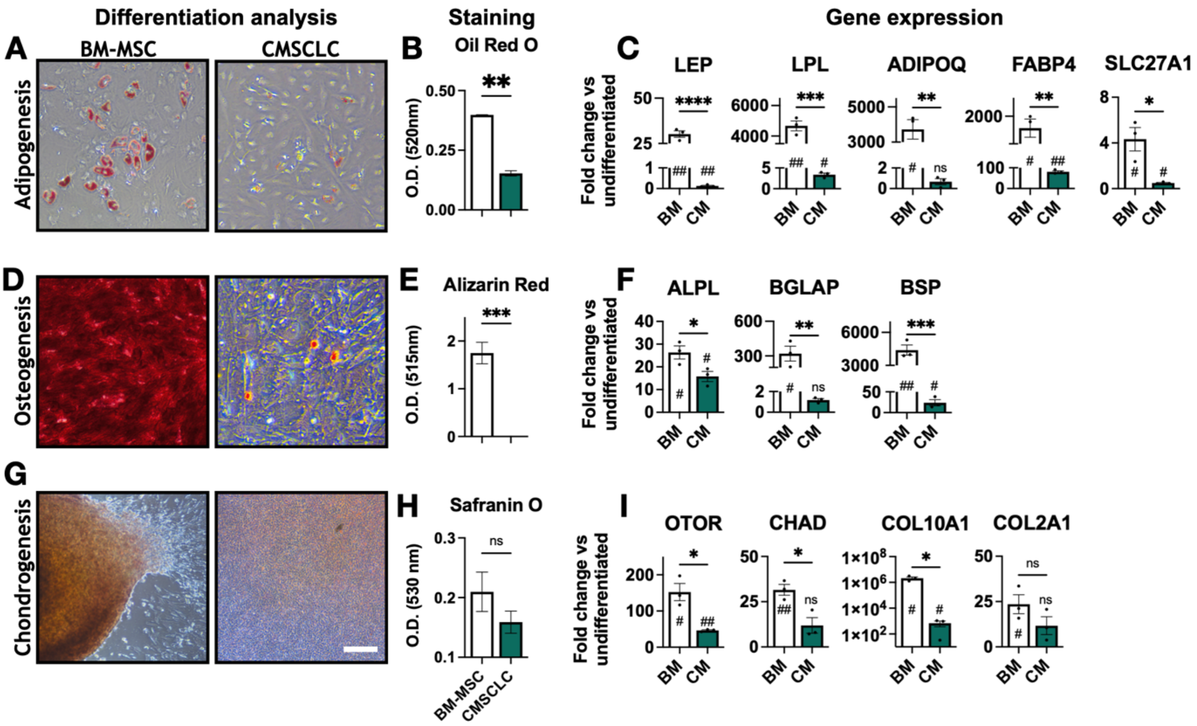

Supplementary Figure 2. **A**. Images of BM-MSCs and CMSCLCs following adipocyte differentiation, stained with Oil Red O. **B**. Quantification of BM-MSC and CMSCLC Oil Red O staining intensity, compared by unpaired *t*-test. **C**. Gene expression of adipose-differentiated BM-MSCs (BM) and CMSCLCs (CM) relative to undifferentiated cells. Comparing pre- to post-differentiation is shown by # (one-way t-test) and BM vs CM is shown by * (unpaired t-test). **D, E, F**. Osteogenesis differentiation. **G, H, I**. Chondrogenesis differentiation. Scale bar 100 µm. * = $P < 0.05$, ** = $P \leq 0.01$, *** = $P \leq 0.001$, **** = $P \leq 0.0001$

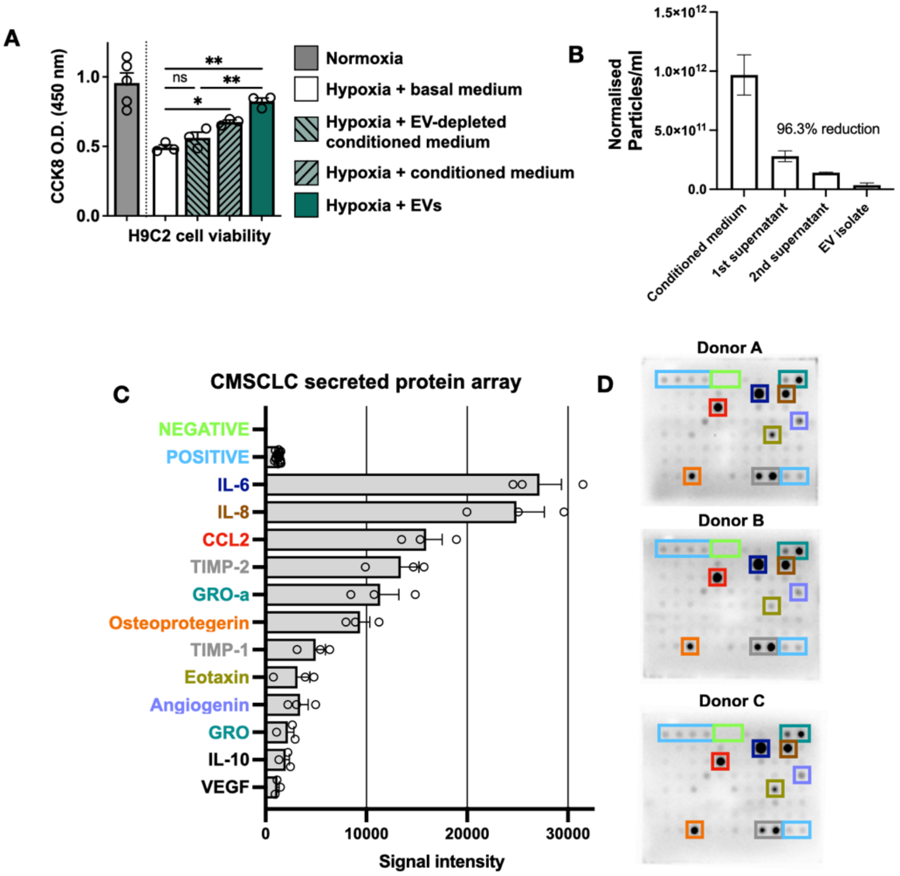

Supplementary Figure 3. (**A**) Viability assay results from rat cardiomyoblast hypoxia experiments. Samples were compared using ANOVA with Tukey's multiple comparison test. ns = not significant, * = P ≤ 0.05, ** = P ≤ 0.01. (**B**) Validation of EV depletion of conditioned medium. Particle counts of original conditioned medium, supernatant following 1 and 2 rounds of ultracentrifugation, and the final EV isolate were measured by NTA. (**C**) Quantified results of cytokine array showing highest detected proteins (n = 3 CMSCLC donors). (**D**) Annotated images of arrays from 3 donors. Coloured boxed correspond to the proteins quantified in (**C**).

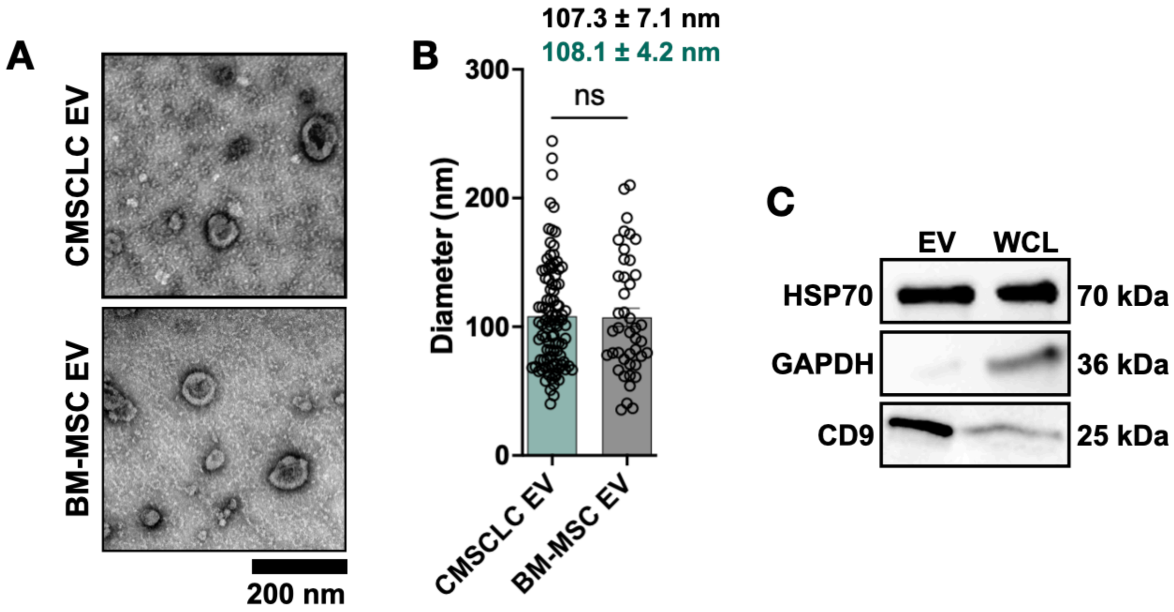

Supplementary Figure 4. (**A**) Conventional TEM images of EVs isolated from CMSCLCs and BM-MSCs. (**B**) Quantification of EV diameter based on cryoEM imaging. ≥ 50 EVs were counted per group, from ≥ 2 separate isolations per group. (**C**) Western blot of HSP70, GAPDH and CD9 in CMSCLC EVs (EV) and CMSCLC whole cell lysates (WCL).

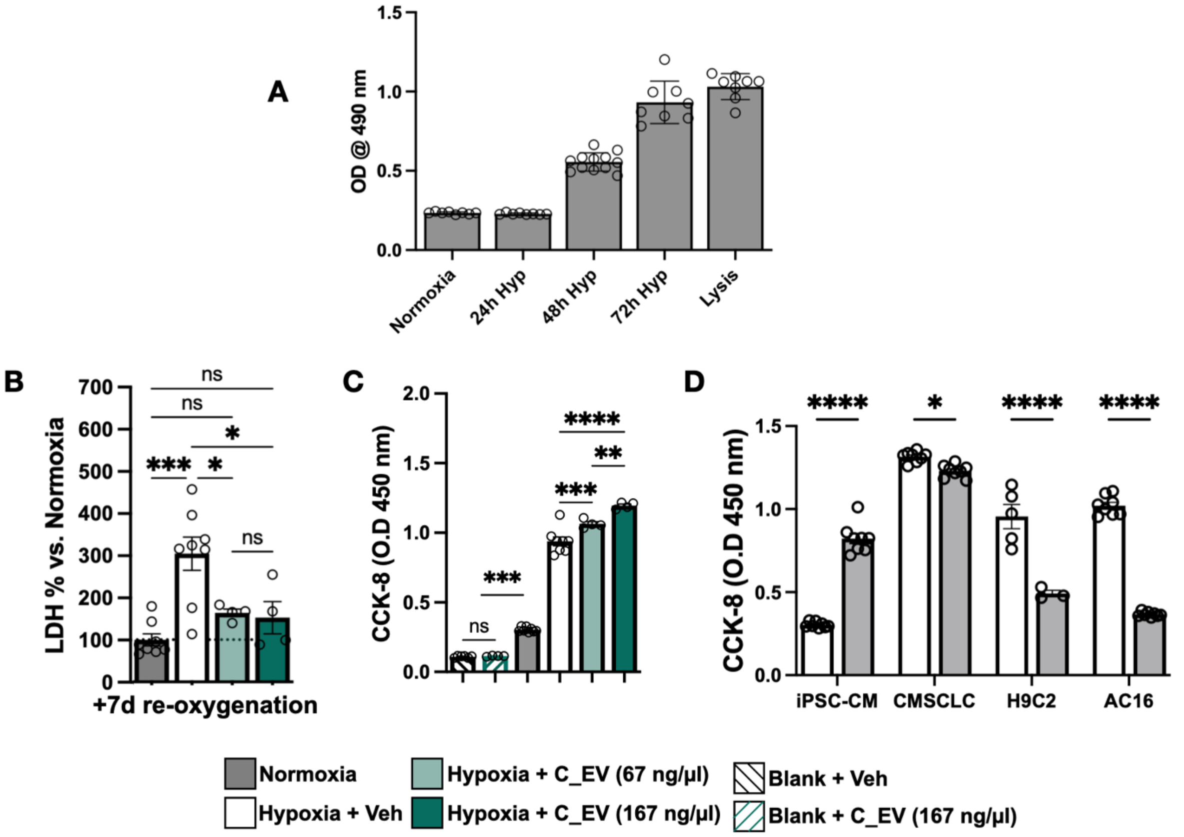

Supplementary Figure 5. (**A**) Culture medium LDH levels after 24/48/72h hypoxia timepoints. Lysed cells, representing maximum LDH release, are also shown. (**B**) Culture medium LDH levels 7 days following re-oxygenation and replacement of medium without EVs, normalised to normoxic cells. Groups were compared by one-way ANOVA with Tukey's multiple comparison test. (**C**) CCK-8 absorbance readings for iPSC-CMs cultured under normoxia, hypoxia with vehicle (Veh) or hypoxia with CMSCLC EVs at 67 ng/µl or 167 ng/µl. OD readings of blank samples with and without EVs are also shown. (**D**) CCK-8 absorbance readings for iPSC-CMs, CMSCLCs and H9C2 cells following culture in normoxia or hypoxia conditions. Each group was compared by unpaired t-test. The annotated comparisons were made by one-way ANOVA. ns = not significant, * = $P < 0.05$, ** =. $P \leq 0.01$, *** = $P \leq 0.001$, **** = $P \leq 0.0001$.

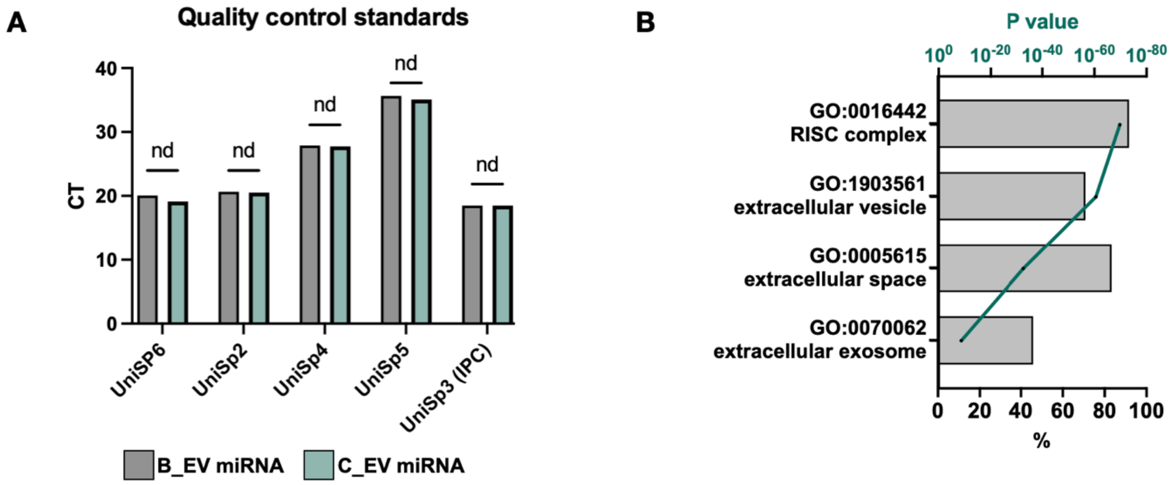

Supplementary Figure 6. (**A**). Quality control data for miRNA array showing cycle threshold (CT) values of spike-in controls. Samples were compared by multiple unpaired t-tests (Benjamini FDR approach). UniSp2/4/5 represent high, medium and low expressed miRNAs respectively. UniSP6 confirms successful miRNA isolation and reverse transcription. UniSp3 shows consistent amplification across multiple miRNA array plates. nd = not a discovery ($P > 0.05$) (**B**) Cellular component gene ontology (GO) predictions for top 50 CMSCLC EVs. The upper X axis shows P values and the lower X axis shows the percentage of top 50 miRNA in each GO.

Term	%	Bonferroni P-value
GO:0035195~gene silencing by miRNA	98.0	1.86653E-72
GO:0035278~miRNA mediated inhibition of translation	46.0	7.49883E-37
GO:0016525~negative regulation of angiogenesis	30.0	1.81686E-17
GO:0010629~negative regulation of gene expression	34.0	5.82828E-15
GO:0035279~mRNA cleavage involved in gene silencing by miRNA	22.0	1.08719E-14
GO:0090051~negative regulation of cell migration involved in sprouting angiogenesis	20.0	3.65707E-13
GO:0050728~negative regulation of inflammatory response	26.0	3.65707E-13
GO:0070104~negative regulation of interleukin-6-mediated signaling pathway	12.0	6.81207E-09
GO:0030336~negative regulation of cell migration	22.0	8.46015E-09
GO:1903588~negative regulation of blood vessel endothelial cell proliferation involved in sprouting angiogenesis	14.0	1.34088E-08
GO:1904707~positive regulation of vascular smooth muscle cell proliferation	16.0	1.85171E-08
GO:0010667~negative regulation of cardiac muscle cell apoptotic process	14.0	6.36730E-08
GO:0045766~positive regulation of angiogenesis	20.0	8.13290E-08
GO:1903589~positive regulation of blood vessel endothelial cell proliferation involved in sprouting angiogenesis	12.0	6.96252E-07
GO:1904995~negative regulation of leukocyte adhesion to vascular endothelial cell	10.0	8.36391E-07
GO:1905563~negative regulation of vascular endothelial cell proliferation	12.0	1.39695E-06
GO:1904046~negative regulation of vascular endothelial growth factor production	12.0	2.11481E-06
GO:1903671~negative regulation of sprouting angiogenesis	12.0	2.56980E-06
GO:0008285~negative regulation of cell proliferation	24.0	4.11475E-06
GO:0032715~negative regulation of interleukin-6 production	14.0	8.35443E-06
GO:1900016~negative regulation of cytokine production involved in inflammatory response	12.0	1.91299E-05
GO:0090370~negative regulation of cholesterol efflux	10.0	1.02957E-04
GO:1904754~positive regulation of vascular associated smooth muscle cell migration	10.0	1.23324E-04
GO:0051897~positive regulation of protein kinase B signaling	14.0	2.73564E-04
GO:0030514~negative regulation of BMP signaling pathway	12.0	3.14398E-04
GO:1904893~negative regulation of STAT cascade	8.0	4.31472E-04
GO:1905064~negative regulation of vascular smooth muscle cell differentiation	8.0	4.31472E-04
GO:0043065~positive regulation of apoptotic process	18.0	4.56840E-04
GO:0070374~positive regulation of ERK1 and ERK2 cascade	16.0	4.60227E-04
GO:0090050~positive regulation of cell migration involved in sprouting angiogenesis	10.0	5.95539E-04
GO:1905205~positive regulation of connective tissue replacement	8.0	8.44416E-04
GO:0043537~negative regulation of blood vessel endothelial cell migration	10.0	1.02986E-03
GO:0060354~negative regulation of cell adhesion molecule production	8.0	2.31071E-03
GO:2000134~negative regulation of G1/S transition of mitotic cell cycle	10.0	4.61928E-03
GO:0045668~negative regulation of osteoblast differentiation	10.0	6.02549E-03
GO:0010628~positive regulation of gene expression	18.0	1.20222E-02
GO:1905111~positive regulation of pulmonary blood vessel remodeling	6.0	1.30184E-02
GO:1904753~negative regulation of vascular associated smooth muscle cell migration	8.0	1.44595E-02
GO:1903672~positive regulation of sprouting angiogenesis	8.0	1.61527E-02

GO:1902430~negative regulation of beta-amyloid formation	8.0	1.79687E-02
GO:0032705~negative regulation of interleukin-21 production	6.0	2.15695E-02
GO:0035924~cellular response to vascular endothelial growth factor stimulus	8.0	2.90088E-02
GO:0032088~negative regulation of NF-kappaB transcription factor activity	10.0	2.94641E-02
GO:0001937~negative regulation of endothelial cell proliferation	8.0	4.35924E-02
GO:1904465~negative regulation of matrix metallopeptidase secretion	6.0	4.46223E-02
GO:1904684~negative regulation of metalloendopeptidase activity	6.0	4.46223E-02

Cellular Component

Term	%	Bonferroni
GO:0016442~RISC complex	96.0	1.29164152736023E-77
GO:1903561~extracellular vesicle	68.0	8.9186973680493E-59
GO:0005615~extracellular space	84.0	2.35870916000317E-33
GO:0070062~extracellular exosome	44.0	9.1527062595631E-08

Molecular Function

Term	%	Bonferroni
GO:1903231~mRNA binding involved in posttranscriptional gene silencing	92.0	5.40125741544182E-84
GO:0003730~mRNA 3'-UTR binding	62.0	5.94737822464547E-46

Supplemental Figure 7. Biological pathway, cellular component and molecular function predictions for top 50 BM-MSC EV miRNAs. Data were generated by DAVID Functional Annotation Tool (https://david.ncifcrf.gov/).

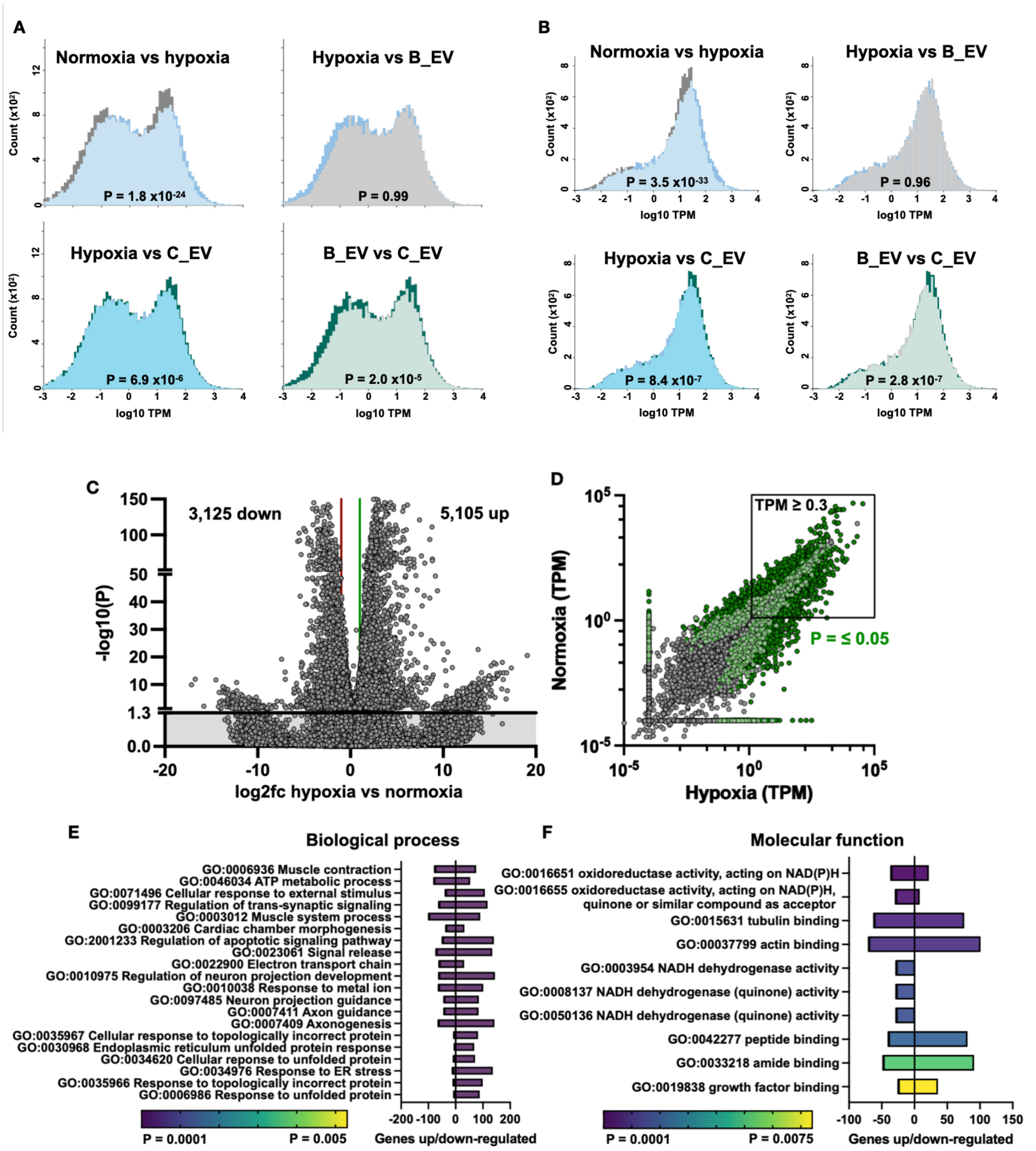

Supplementary Figure 8. (**A**) Comparison of all gene TPM distribution for selected sample pairs. Groups were compared by Kolmogorov-Smirnov test. P-values are indicated. (**B**) Comparison of protein coding gene TPM distribution for selected sample pairs. Groups were compared by Kolmogorov-Smirnov test. P-values are indicated. (**C**). Volcano plot of hypoxia + vehicle vs. normoxia group. (**D**) Scatter plot of hypoxia + vehicle vs. normoxia group. (**E**) Pyramid plot of gene ontology biological process. (**F**). Pyramid plot of gene ontology molecular function. Significance is shown by the colour scale.

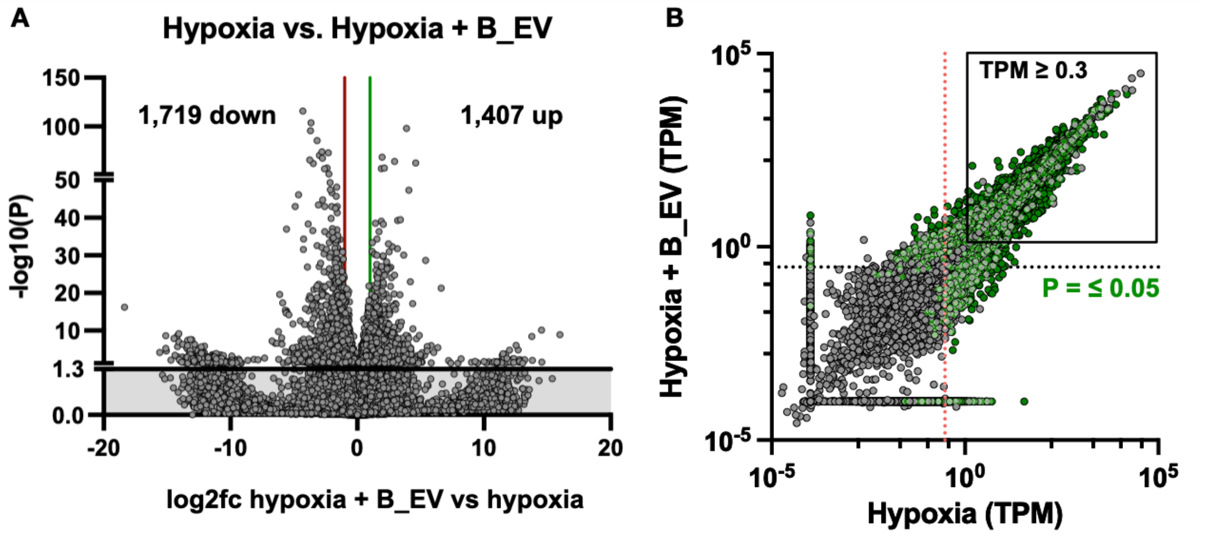

C

Molecular Function

ID	Description	GeneRatio	Up/down	BgRatio	p.adjust
GO:0022836	gated channel activity	80/2031	28/52	341/18432	3.81E-08
GO:0015267	channel activity	97/2031	38/59	494/18432	3.79E-06
GO:0022803	passive transmembrane transporter activity	97/2031	38/59	495/18432	3.79E-06
GO:0005216	ion channel activity	89/2031	32/57	446/18432	4.77E-06
GO:0005261	cation channel activity	72/2031	29/43	346/18432	1.58E-05
GO:0046873	metal ion transmembrane transporter activity	85/2031	28/57	436/18432	1.96E-05
GO:0022843	voltage-gated cation channel activity	37/2031	12/25	151/18432	0.000323
GO:0005244	voltage-gated ion channel activity	45/2031	14/31	201/18432	0.000323
GO:0022832	voltage-gated channel activity	45/2031	14/31	201/18432	0.000323
GO:0005249	voltage-gated potassium channel activity	27/2031	10/17	99/18432	0.000538
GO:0005267	potassium channel activity	31/2031		122/18432	0.000538
GO:0015079	potassium ion transmembrane transporter activity	37/2031		158/18432	0.000538
GO:0015276	ligand-gated ion channel activity	35/2031		146/18432	0.000538
GO:0022834	ligand-gated channel activity	35/2031		146/18432	0.000538
GO:0019838	growth factor binding	32/2031		132/18432	0.000962
GO:0099094	ligand-gated cation channel activity	29/2031		116/18432	0.001213
GO:0035254	glutamate receptor binding	15/2031		42/18432	0.00145
GO:0008017	microtubule binding	53/2031		271/18432	0.00145
GO:0005178	integrin binding	35/2031		156/18432	0.001823
GO:0004016	adenylate cyclase activity	7/2031		11/18432	0.002473

KEGG

ID	Description	GeneRatio	Up/down	BgRatio	p.adjust
hsa04020	Calcium signaling pathway - Homo sapiens (human)	60/960	27/33	253/8621	2.17E-06
hsa04024	cAMP signaling pathway - Homo sapiens (human)	47/960	22/25	225/8621	0.001563
hsa04971	Gastric acid secretion - Homo sapiens (human)	22/960	13/9	76/8621	0.001563
hsa04970	Salivary secretion - Homo sapiens (human)	25/960	10/15	93/8621	0.001563
hsa04750	Inflammatory mediator regulation of TRP channels - Homo sapiens	25/960	14/11	98/8621	0.003254
hsa00260	Glycine, serine and threonine metabolism - Homo sapiens (human)	14/960	7/7	40/8621	0.003254
hsa05414	Dilated cardiomyopathy - Homo sapiens (human)	24/960	14/10	96/8621	0.004648
hsa04540	Gap junction - Homo sapiens (human)	22/960	13/9	88/8621	0.007842
hsa04080	Neuroactive ligand-receptor interaction - Homo sapiens (human)	63/960	25/38	367/8621	0.008441
hsa04270	Vascular smooth muscle contraction - Homo sapiens (human)	29/960	15/14	134/8621	0.008441

Supplementary Figure 9. (**A**) Volcano and (**B**) scatter plots of hypoxia + vehicle compared to hypoxia + BM-MSC EV (B_EV). The tables show the highest differentially expressed gene ontology groups for molecular function and KEGG.

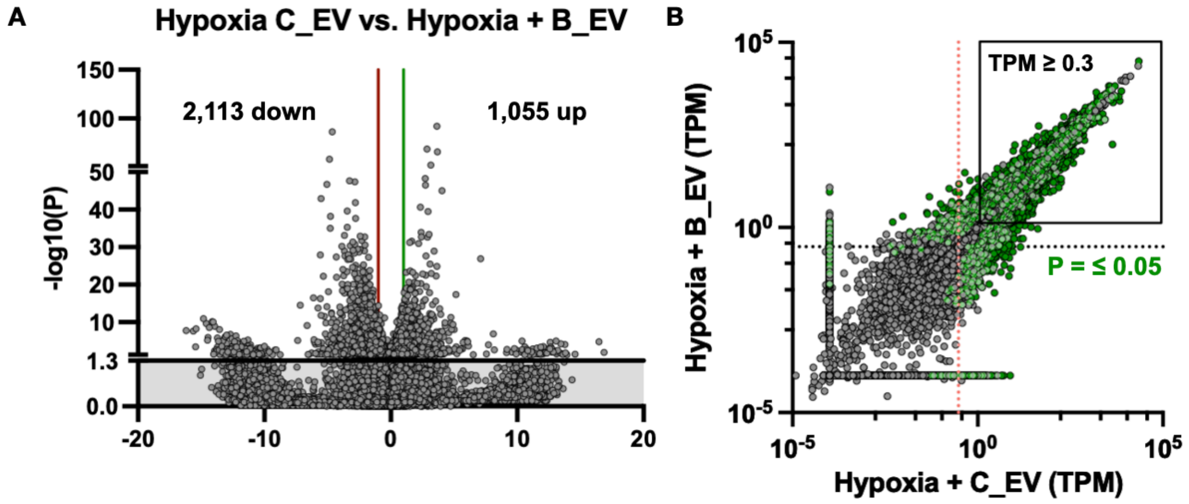

C

Molecular Function

ID	Description	GeneRatio	BgRatio	pvalue	p.adjust
GO:0022836	gated channel activity	81/2101	341/18432	7.03E-11	7.89E-08
GO:0015267	channel activity	101/2101	494/18432	2.85E-09	9.77E-07
GO:0022803	passive transmembrane transporter activity	101/2101	495/18432	3.18E-09	9.77E-07
GO:0019838	growth factor binding	40/2101	132/18432	3.58E-09	9.77E-07
GO:0005216	ion channel activity	93/2101	446/18432	4.36E-09	9.77E-07
GO:0046873	metal ion transmembrane transporter activity	91/2101	436/18432	6.09E-09	1.14E-06
GO:0005261	cation channel activity	74/2101	346/18432	5.61E-08	8.99E-06
GO:0004970	ionotropic glutamate receptor activity	12/2101	19/18432	1.09E-07	1.53E-05
GO:0008066	glutamate receptor activity	14/2101	27/18432	2.83E-07	3.53E-05
GO:0099094	ligand-gated cation channel activity	32/2101	116/18432	1.33E-06	0.000149
GO:0015291	secondary active transmembrane transporter activity	54/2101	246/18432	1.47E-06	0.00015
GO:0015276	ligand-gated ion channel activity	37/2101	146/18432	1.97E-06	0.00017
GO:0022834	ligand-gated channel activity	37/2101	146/18432	1.97E-06	0.00017
GO:0005244	voltage-gated ion channel activity	46/2101	201/18432	2.63E-06	0.000196
GO:0022832	voltage-gated channel activity	46/2101	201/18432	2.63E-06	0.000196
GO:0005201	extracellular matrix structural constituent	41/2101	173/18432	3.58E-06	0.000251
GO:0022843	voltage-gated cation channel activity	37/2101	151/18432	4.62E-06	0.000305
GO:0005539	glycosaminoglycan binding	51/2101	237/18432	5.19E-06	0.000324
GO:1901681	sulfur compound binding	56/2101	270/18432	5.99E-06	0.000354
GO:0005342	organic acid transmembrane transporter activity	39/2101	166/18432	7.64E-06	0.000429

Supplementary Figure 10. (**A**) Volcano and (**B**) scatter plots of hypoxia + CMSCLC EV (C_EV) compared to hypoxia + BM-MSC EV (B_EV). The tables show the highest differentially expressed gene ontology groups for molecular function.

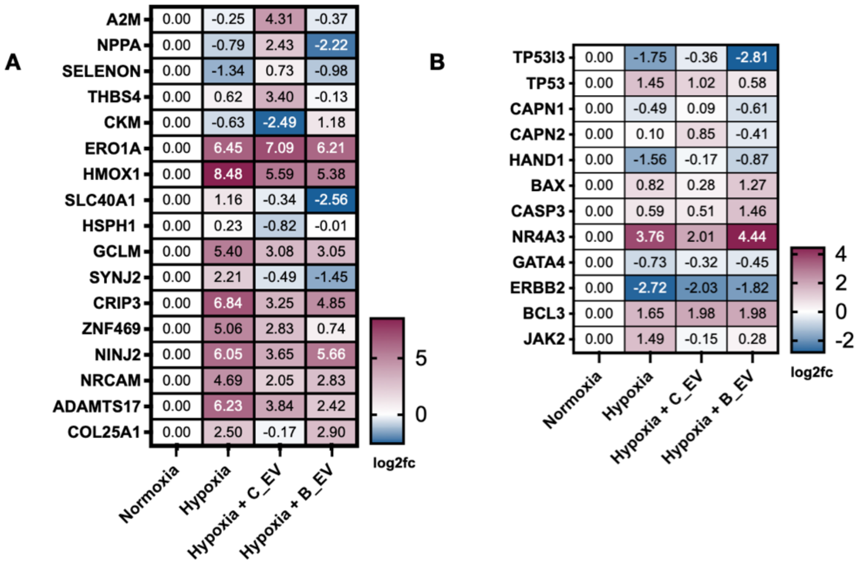

Supplementary Figure 11. (**A**) Summary of highly differentially expressed genes and (**B**) apoptosis-related genes. Numbers show log2fc compared to normoxic iPSC-CMs.

A. Down-regulated C_EV miRNA targets

Gene	Description	Norm TPM	Hypoxia TPM	C_EV TPM	B_EV TPM	FC Norm/ Hyp	FC Hyp/ C_EV	FC Hyp/ B_EV	P Hyp/C_EV	% of top 50 C_EV mirnas
**GCLM**	glutamate-cysteine-ligase-modifier-subunit	1.869	79.483	15.955	15.869	5.410	-2.317	-2.324	2.660E-60	10
**EGR1**	early-growth-response-1	10.926	67.694	16.677	114.792	2.631	-2.021	0.762	1.750E-03	6
**MAP2**	microtubule-associated-protein-2	1.176	104.744	26.706	27.171	6.476	-1.972	-1.947	5.820E-40	2
**KITLG**	KIT-ligand	6.424	86.945	26.502	8.116	3.759	-1.714	-3.421	1.630E-20	14
**JAK2**	Janus-kinase-2	25.338	71.450	23.391	31.108	1.496	-1.611	-1.200	8.420E-07	2
**DSEL**	dermatan-sulfate-epimerase-like	4.273	13.481	4.548	1.907	1.658	-1.568	-2.821	9.565E-17	16
**TXNRD1**	thioredoxin-reductase-1	39.714	1076.187	466.812	336.652	4.760	-1.205	-1.677	1.060E-23	12
**DDAH1**	dimethylarginine-dimethylaminohydrolase-1	12.121	65.401	30.263	7.590	2.432	-1.112	-3.107	1.940E-13	2
**UNKL**	unk-like-zinc-finger	10.526	130.127	61.068	32.418	3.628	-1.091	-2.005	1.070E-17	18

B

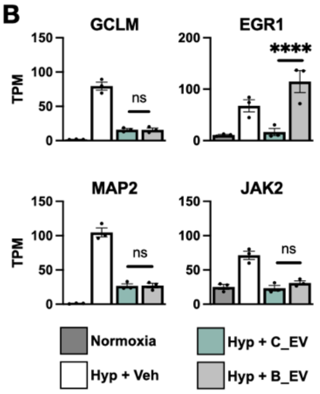

C. Down-regulated B_EV miRNA targets

Gene	Description	Norm TPM	Hypoxia TPM	C_EV TPM	B_EV TPM	FC Norm/ Hyp	FC Hyp/ C_EV	FC Hyp/ B_EV	P Hyp/C_EV	% of top 50 B_EV mirnas
**KITLG**	KIT-ligand	6.424	86.945	26.502	8.116	3.759	-1.714	-3.421	2.577E-19	10
**DDAH1**	dimethylarginine-dimethylaminohydrolase-1	12.121	65.401	30.263	7.590	2.432	-1.112	-3.107	1.934E-15	10
**DDR2**	discoidin-domain-receptor-tyrosine-kinase-2	3.626	38.516	34.868	4.694	3.409	-0.144	-3.037	1.018E-20	4
**SERPINE2**	serpin-family-E-member-2	2.173	96.551	154.019	12.066	5.473	0.674	-3.000	3.092E-25	2
**DSEL**	dermatan-sulfate-epimerase-like	4.273	13.481	4.548	1.907	1.658	-1.568	-2.821	3.884E-15	10
**SERPINE1**	serpin-family-E-member-1	2.221	243.077	115.765	42.050	6.774	-1.070	-2.531	9.145E-08	20
**AMOTL2**	angiomotin-like-2	63.084	182.851	166.320	35.165	1.535	-0.137	-2.378	9.828E-55	16
**GCLM**	glutamate-cysteine-ligase-modifier-subunit	1.869	79.483	15.955	15.869	5.410	-2.317	-2.324	8.522E-24	12
**SLC9A1**	solute-carrier-family-9-member-A1	15.130	45.433	39.495	9.350	1.586	-0.202	-2.281	4.047E-70	10
**ARL4C**	ADP-ribosylation-factor-like-GTPase-4C	2.577	9.240	7.859	1.950	1.842	-0.234	-2.244	3.043E-12	16

D

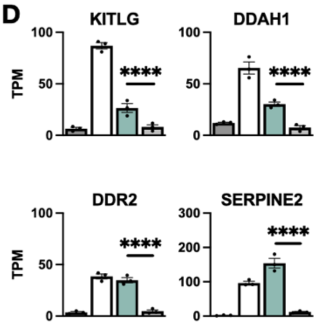

Supplementary Figure 12 (**A, B**) Table and graphs showing top 50 miRNA target predicted genes which were upregulated ≥ 2-fold by hypoxia compared to normoxia, and subsequently downregulated ≥ 2-fold by C_EVs or (**C,D**) B_EVs. The expression level (TPM) for each group is shown, as are log2-fold changes between groups. The percentage of the top 50 miRNAs known to target each gene is shown. Graphs show the expression level of key genes and statistical annotations are based on RNA-seq data adjusted P values. ns = not significant, **** = P ≤ 0.0001.

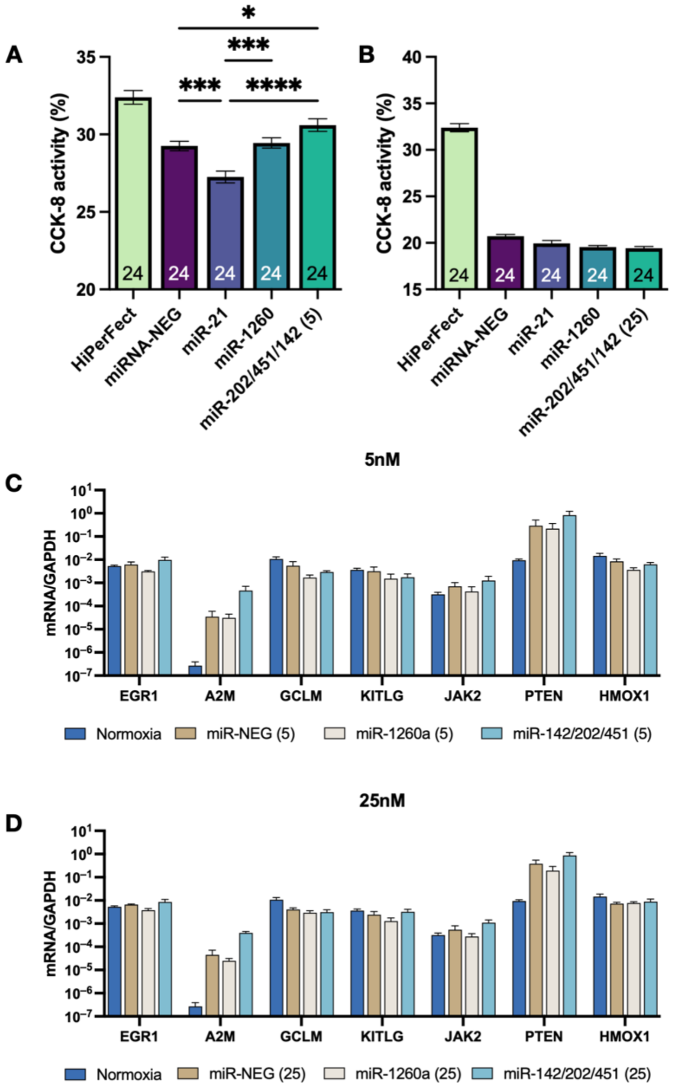

Supplementary Figure 13 (A) CCK-8 activity of human cardiomyocytes under hypoxia incubated with transfection reagent (HiPerFect, 6 nl/µl) only, or transfected with 5 nM negative control (miRNA-NEG), miR-21-5p, miR-1260a or miR-202-5p, miR-451a and miR-142-3p (1.67 nM each). Viability is shown relative to normoxia. (**B**) 25 nM of each mimic was used with transfection reagent at the same concentration. Samples were compared to miRNA-NEG by Dunnett's multiple comparison test and significant results are indicated. * = $P < 0.05$, *** = $P \leq 0.001$, **** = $P \leq 0.0001$. (**C, D**) Gene expression levels of hypoxic cardiomyocyte following incubation with 5 nM or 25 nM miRNA mimics, normalised to GAPDH. The Y axis is presented on a log scale.
