# Supplementary material for: Extracellular vesicles from human cardiac stromal cells up-regulate cardiomyocyte protective responses to hypoxia
Source: Stem Cell Res Ther. 2024 Oct 12;15:363. doi: 10.1186/s13287-024-03983-y (PMC11470622; doi:10.1186/s13287-024-03983-y)
Supplement: Supplementary file 2 — Supplementary Material 2 [file 13287_2024_3983_MOESM2_ESM.docx]

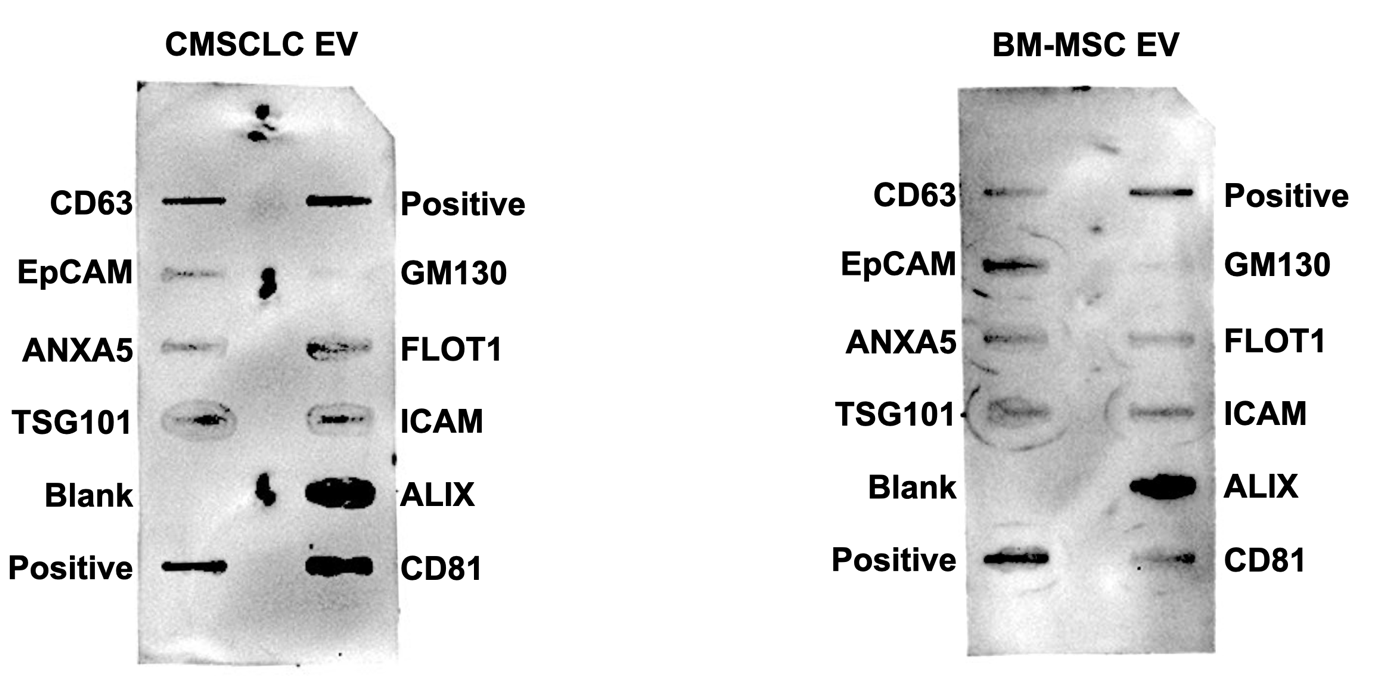


Original uncropped antibody array membrane images, from Figure 2D


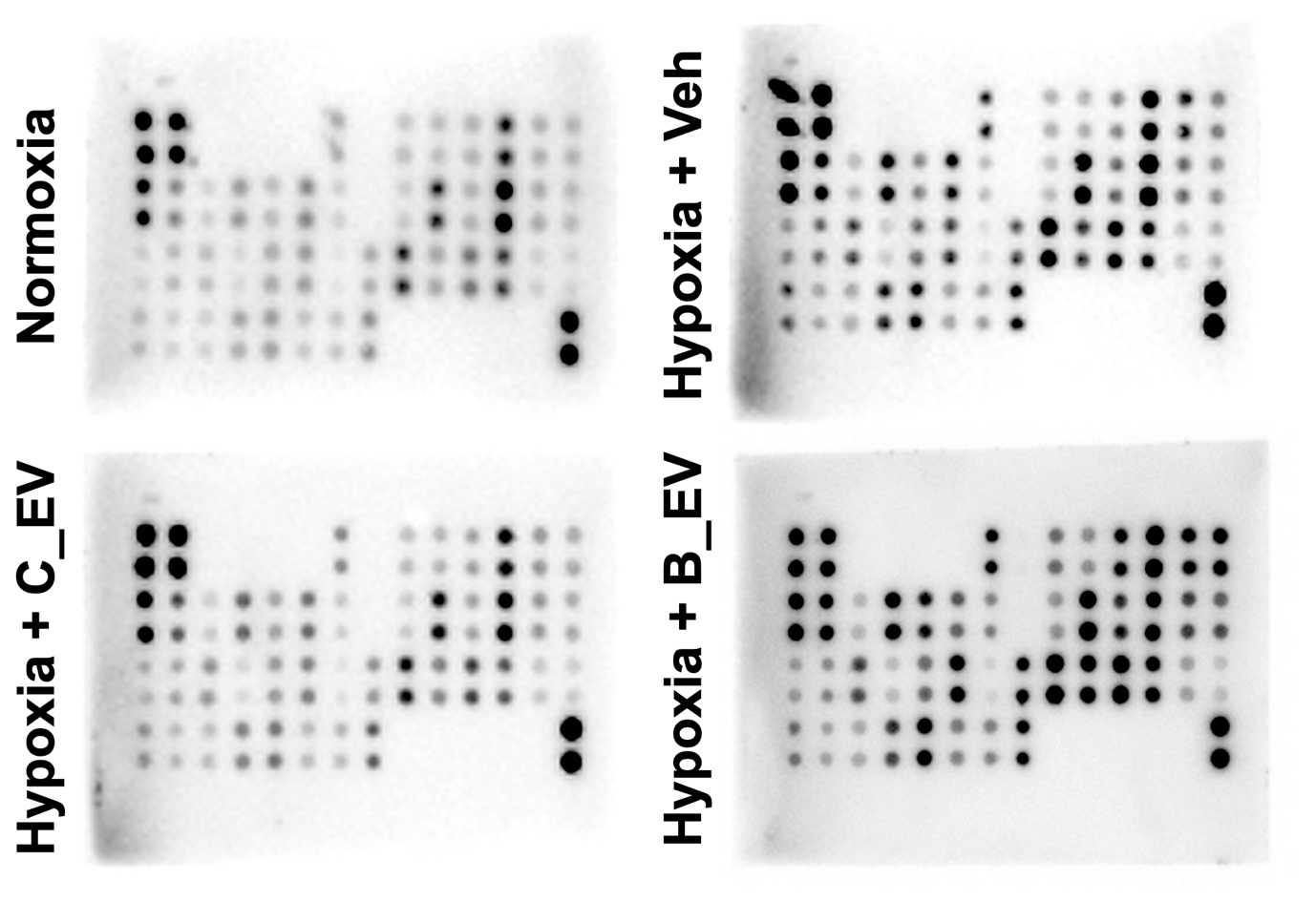


Uncropped antibody arrays from Figure 3D
